# UBE2D3 facilitates NHEJ by orchestrating ATM signalling through multi-level control of RNF168

**DOI:** 10.1038/s41467-024-49431-6

**Published:** 2024-06-12

**Authors:** Zeliha Yalçin, Shiu Yeung Lam, Marieke H. Peuscher, Jaco van der Torre, Sha Zhu, Prasanna V. Iyengar, Daniel Salas-Lloret, Inge de Krijger, Nathalie Moatti, Ruben van der Lugt, Mattia Falcone, Aurora Cerutti, Onno B. Bleijerveld, Liesbeth Hoekman, Román González-Prieto, Jacqueline J. L. Jacobs

**Affiliations:** 1 Division of Oncogenomics, The Netherlands Cancer Institute, Plesmanlaan 121, 1066 CX Amsterdam, the Netherlands; 2 Department of Cell and Chemical Biology, Leiden University Medical Center, Einthovenweg 20, 2333 ZC Leiden, the Netherlands; 3 Proteomics Facility, The Netherlands Cancer Institute, Plesmanlaan 121, 1066 CX Amsterdam, the Netherlands; 4 Andalusian Center for Molecular Biology and regenerative Medicine (CABIMER), Universidad de Sevilla-CSIC-Universidad-Pablo de Olavide, Sevilla, Spain; 5 Departamento de Biología Celular, Facultad de Biología, Universidad de Sevilla, Sevilla, Spain

**Keywords:** Non-homologous-end joining, Telomeres

## Abstract

Maintenance of genome integrity requires tight control of DNA damage response (DDR) signalling and repair, with phosphorylation and ubiquitination representing key elements. How these events are coordinated to achieve productive DNA repair remains elusive. Here we identify the ubiquitin-conjugating enzyme UBE2D3 as a regulator of ATM kinase-induced DDR that promotes non-homologous end-joining (NHEJ) at telomeres. UBE2D3 contributes to DDR-induced chromatin ubiquitination and recruitment of the NHEJ-promoting factor 53BP1, both mediated by RNF168 upon ATM activation. Additionally, UBE2D3 promotes NHEJ by limiting RNF168 accumulation and facilitating ATM-mediated phosphorylation of KAP1-S824. Mechanistically, defective KAP1-S824 phosphorylation and telomeric NHEJ upon UBE2D3-deficiency are linked to RNF168 hyperaccumulation and aberrant PP2A phosphatase activity. Together, our results identify UBE2D3 as a multi-level regulator of NHEJ that orchestrates ATM and RNF168 activities. Moreover, they reveal a negative regulatory circuit in the DDR that is constrained by UBE2D3 and consists of RNF168- and phosphatase-mediated restriction of KAP1 phosphorylation.

## Abstract (web summary)

Ubiquitin system factors are critical regulators of DNA repair pathways. Through a functional genetic screen, the authors identify a novel role of ubiquitin-conjugating enzyme UBE2D3 in promoting DNA repair at deprotected telomeres by limiting the accumulation of RNF168 and promoting ample ATM-dependent phosphorylation of KAP1.

## Introduction

Numerous cancers are associated with DNA repair defects^1,2^, but the (epi)genetic causes underlying many such cases remain elusive. Poor predictability of the sensitivity of cancers to DNA-damaging anti-cancer treatments and the meagre understanding of therapy resistance further highlight the need for identifying critical factors controlling DNA repair. To enable this we previously developed an approach to identify factors contributing to genomic instability following NHEJ-mediated fusion of deprotected telomeres^3^. This approach relies on inactivation of the shelterin component TRF2 in *Trf2* ^*-/-*^*;p53* ^*-/-*^ mouse embryonic fibroblasts (MEFs) expressing a temperature-sensitive TRF2 allele (I468A; TRF2ts)^3,4^. TRF2ts MEFs have intact telomere protection at the permissive temperature (32 °C) when TRF2ts functionally substitutes for the absence of endogenous TRF2 but lose this protection at non-permissive temperatures (37–39 °C) when TRF2ts cannot bind telomeres. This induces an ATM-mediated DNA damage response (DDR) at telomeres that results in NHEJ-dependent telomere fusion and cell death due to genomic crisis. Inhibition of factors required for efficient NHEJ allows cells with TRF2 inactivation to avoid this crisis. Hence in this setting, cell survival in spite of telomere deprotection serves as a proxy for NHEJ-deficiency. Through this approach we previously identified MAD2L2 (also known as REV7) as a critical factor in DNA repair pathway choice and uncovered the roles of RNF8 and MMSET in telomere NHEJ^3,5,6^.

We now report the identification of the ubiquitin-conjugating (E2) enzyme UBE2D3 (or UBCH5C) as a regulator of NHEJ at telomeres, and addressed how UBE2D3 acts in the DDR. We uncovered that UBE2D3 controls the activity of RNF168, a ubiquitin-ligase (or E3 enzyme) with critical roles in the DDR^7–9^, in multiple independent ways. On the one hand UBE2D3 contributes to degradation of RNF168, resulting in RNF168 hyperaccumulation in the absence of UBE2D3. On the other hand, UBE2D3 contributes to chromatin ubiquitination and 53BP1 recruitment that are mediated by RNF168 upon ATM kinase activation by DNA damage^10^, such that in the absence of UBE2D3, chromatin ubiquitination and 53BP1 recruitment are impaired, despite RNF168 being stabilised. Importantly, we found UBE2D3-deficiency and associated RNF168 hyperaccumulation to reduce ATM-dependent KAP1-S824 phosphorylation, and thereby NHEJ, which at least in part appeared attributable to aberrant PP2A phosphatase activity. This reveals the existence of a negative feedback regulation between ubiquitin and kinase signalling in the DDR in which ATM kinase activity towards the chromatin factor KAP1 is counteracted by phosphatase activity, in a manner that is controlled by UBE2D3 and RNF168. The latter itself being recruited to sites of DNA damage upon ATM activation.

## Results

### UBE2D3 promotes NHEJ and genome instability following telomere deprotection

**Fig. 1:**
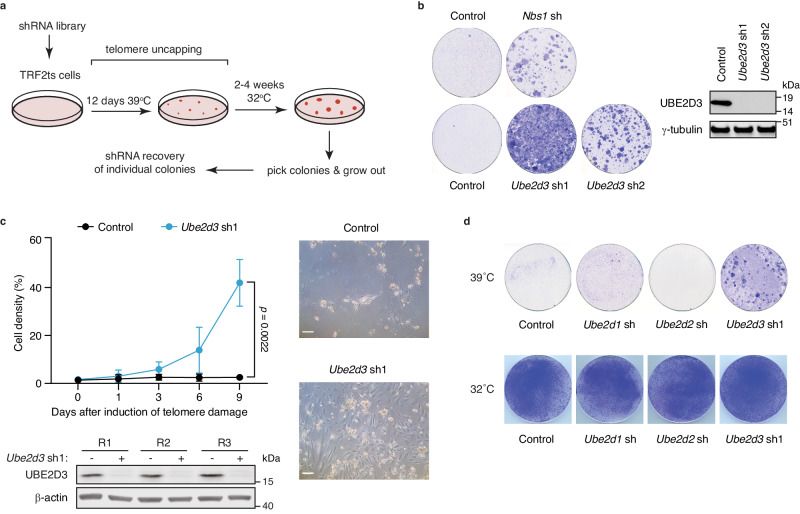
UBE2D3 is a regulator of telomere-driven genomic instability. **a** Outline of the screen performed to identify factors controlling telomere-driven genomic instability. **b** Survival assays after 2 weeks of telomere uncapping for 2 independent *Ube2d3* shRNAs and 1 *Nbs1* shRNA. Representative plates from 8 independent experiments for control and *Ube2d3* sh1 and 2 independent experiments for *Ube2d3* sh2 and *Nbs1* sh are shown. Representative immunoblots for *Ube2d3* depletion from 2 independent experiments are shown. **c** Left: Growth curves of control and *Ube2d3* knockdown TRF2ts MEFs (clone C15) upon induction of telomere uncapping (*n* = *3* independent experiments; mean ± SEM; two-tailed Student’s *t*-test). Immunoblot for UBE2D3 in cells used for growth assays are shown below (R1, R2, R3 = replicate 1, 2, 3). Right: Representative photographs of arrested and dying control TRF2ts MEFs and proliferating *Ube2d3* knockdown TRF2ts MEFs after 12 days of telomere uncapping (TRF2ts clone B17). Scale bar represents 10 μm. Growth curves from these cells can be found in Supplementary Fig. 1c. **d** Survival assays upon *Ube2d1*, *Ube2d2* and *Ube2d3* depletion, after 12 days of telomere uncapping and at 32 °C. Representative plates from 2 independent experiments are shown. Source data are provided as a Source Data file.

During initial assessment of the feasibility of the TRF2ts approach for functional genetic screening we infected TRF2ts MEFs with an NKI mouse shRNA library that targets ~15,000 mouse genes with 2 independent shRNAs per gene^11^. Cells were kept at 39 °C for 12 days to induce telomere uncapping and lethal genomic crisis due to telomeric NHEJ. Subsequently, cells were returned to 32 °C to restore TRF2 activity and allow surviving cells to form colonies. Several individual colonies were isolated and expanded, and shRNA sequences were recovered by PCR on genomic DNA and Sanger sequencing (Fig. 1a). Among the individual shRNA inserts, we recovered an shRNA targeting the MRN-complex component NBS1, known to contribute to telomere end-joining^12,13^. Indeed, inhibition of NBS1 with this shRNA resulted in rescue from lethal telomere-induced genomic instability (Fig. 1b).

Unexpectedly, we also recovered an shRNA targeting the ubiquitin-conjugating (E2) enzyme UBE2D3. UBE2D3 is known to have promiscuous activity in vitro, but its in vivo roles are less well defined^14^, with no known roles at uncapped telomeres or in NHEJ. Knockdown of *Ube2d3* with multiple independent shRNAs markedly increased the survival of TRF2ts cells subjected to prolonged telomere uncapping, without affecting the levels of DNA ligase IV, the critical DNA ligase in classical NHEJ at telomeres, or of TRF2ts itself (Fig. 1b, c and Supplementary Fig. 1a–c). In addition, we confirmed that *Ube2d3* shRNA1 specifically targets *Ube2d3* and does not reduce expression of the highly related UBE2D family members *Ube2d1* and *Ube2d2*, or of *Ubc13*, another DDR E2 enzyme known to act with E3 enzymes previously implicated in telomere-driven genomic instability^5,8,15–17^ (Supplementary Fig. 1d). Moreover, complementation with exogenous shRNA-resistant UBE2D3 abolished the survival of UBE2D3-depleted cells subjected to telomere uncapping (Supplementary Fig. 1e–g). Together, this indicates an important role for UBE2D3 in telomere-induced genomic instability.

Neither depletion of UBE2D1 nor UBE2D2 phenocopied UBE2D3 depletion in causing MEFs to survive telomere uncapping (Fig. 1d and Supplementary Fig. 1h). This suggests specificity among UBE2D family members and identifies UBE2D3 as the main UBE2D family member contributing to telomere-driven genomic crisis in MEFs. This could reflect differences in expression, as UBE2D3 is the most or highest expressed family member (as reported^15^ and according to Human Protein Atlas: www.proteinatlas.org). Yet, while ectopic expression of UBE2D3 abolished the survival of TRF2ts cells depleted for endogenous UBE2D3 and subjected to telomere uncapping (39 °C), overexpression of UBE2D1 in UBE2D3-depleted cells did not specifically impair the survival of such cells under telomere uncapping conditions (39 °C) and already reduced cell viability under unchallenged conditions (32 °C) (Supplementary Fig. 1e–g). Thus, we did not detect significant functional redundancy among UBE2D family members in telomere uncapping responses (see also further down).

**Fig. 2:**
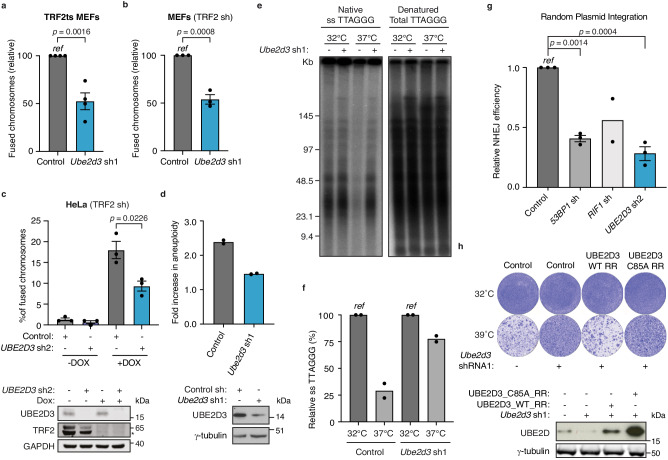
UBE2D3 promotes NHEJ at uncapped telomeres and in a wider genomic context. **a** Quantification of chromosome fusions in TRF2ts cells transduced as indicated upon 24 h of telomere uncapping at 39 °C (*n* = *4* independent experiments; mean ± SEM; two-tailed Student’s *t*-test). **b** Quantification of chromosome fusions in control and UBE2D3-depleted SV40-immortalised wild-type (WT) MEFs, at 6 days after *Trf2* shRNA expression (*n* = *3* independent experiments; mean ± SEM; two-tailed Student’s *t*-test). **c** Top: Quantification of chromosome fusions in control and UBE2D3-depleted HeLa cells expressing a doxycycline-inducible *TRF2* shRNA (*n* = *3* independent experiments; mean ± SEM; two-tailed Student’s *t*-test). Bottom: Immunoblotting for TRF2 and UBE2D3 in cells used for chromosome fusion analysis. The asterisk indicates TRF2. Representative blots from 3 independent experiments. **d** Top: Quantification of aneuploidy (>4N DNA content) based on DNA content analysis by flow cytometry of TRF2ts MEFs, transduced as indicated, subjected to telomere uncapping and stained with propidium iodide (*n* = *2* independent experiments). Bottom: Immunoblotting for UBE2D3 in cells used for aneuploidy assays. Representative blots from 2 independent experiments. **e** Telomeric G-overhang signal in control and UBE2D3-depleted TRF2ts MEFs at 32 °C and after 48 h of telomere uncapping. Left: Native conditions to detect single-strand (ss) TTAGGG repeats. Right: Denaturing conditions for total telomeric DNA. **f** Quantification of relative telomeric G-overhang signal (*n* = *2* independent experiments). **g** NHEJ-mediated repair in U2OS cells transduced as indicated and analysed for random plasmid integration. ShRNAs against the NHEJ promoting factors 53BP1 and RIF1 serve as positive controls (mean ± SEM of *n* = *3* independent experiments for *53BP1* sh and *UBE2D3* sh2 and *n* = *2* independent experiments for *RIF1* sh are shown). Statistical significance was calculated using one-way analysis of variance (ANOVA) with Tukey’s multiple comparisons test. **h** Top: Survival assays of TRF2ts cells depleted for UBE2D3 with *Ube2d3* shRNA1 and complemented with RNAi-resistant (RR) wild-type UBE2D3 (LZRS_UBE2D3_WT_RR) and UBE2D3 C85A (LZRS_UBE2D3_C85A_RR). Representative plates from 3 independent experiments. Bottom: Western blot analysis of *Ube2d3* depletion, and UBE2D3 RR and UBE2D3 C85A RR expression in TRF2ts MEFs used in (**h**). Representative blots from 3 independent experiments. *Ref* reference. Source data are provided as a Source Data file.

Consistent with UBE2D3 contributing to telomere uncapping-induced genomic crisis, UBE2D3-depleted TRF2ts MEFs showed a ±50% reduction in NHEJ-mediated telomere fusion (Fig. 2a and Supplementary Fig. 2a). Also, wild-type MEFs, human HeLa cells or human BJ fibroblasts depleted for UBE2D3 were significantly impaired in telomere fusion after shRNA-mediated knockdown of TRF2 (Fig. 2b, c and Supplementary Fig. 2b, c). Thus, UBE2D3 facilitates NHEJ at uncapped telomeres in both mouse and human cells and independent of the method of TRF2 inhibition. In line with UBE2D3 promoting telomere fusions that disturb chromosome segregation, UBE2D3 depletion prevented aneuploidy after telomere uncapping (Fig. 2d and Supplementary Fig. 2d). Furthermore, while telomeric 3’ G-overhangs are degraded in a DNA ligase IV- and NHEJ-dependent manner following loss of TRF2-mediated telomere protection^18,19^, UBE2D3-depleted cells retained telomeric 3’ G-overhangs (Fig. 2e, f). This further illustrates that in the absence of UBE2D3 uncapped telomeres are not efficiently processed by the NHEJ machinery. In addition, UBE2D3-depleted cells were also impaired in NHEJ-mediated random plasmid integration, indicating a role for UBE2D3 in NHEJ beyond telomeres, in a wider DNA repair context (Fig. 2g and Supplementary Fig. 2e).

### UBE2D3 contributes to DDR-induced chromatin ubiquitination

As the engagement of the competing NHEJ and homologous recombination DNA repair pathways is cell cycle regulated^20^, we addressed whether changes in cell cycle progression might underlie the reduced NHEJ in UBE2D3-depleted cells. Importantly, UBE2D3 depletion did not affect cell cycle distribution at the permissive temperature, nor after 12 h of telomere uncapping (Supplementary Fig. 3a–c), indicating that UBE2D3 promotes NHEJ independent of cell cycle regulation. Next, we addressed the importance of the catalytic activity of UBE2D3 for NHEJ-driven genomic crisis. UBE2D3-depleted TRF2ts cells restored with exogenous wild-type UBE2D3 underwent lethal telomere NHEJ-driven genomic crisis, similar to cells with unperturbed UBE2D3. On the contrary, UBE2D3-depleted cells complemented with catalytically inactive mutant UBE2D3 (C85A) retained the ability to survive telomere uncapping, similar to UBE2D3-depleted cells with impaired NHEJ at telomeres (Fig. 2h). This indicates that the E2 enzyme activity of UBE2D3, disrupted by the C85A mutation, is essential for efficient NHEJ-mediated ligation of uncapped telomeres that results in genomic crisis.

**Fig. 3:**
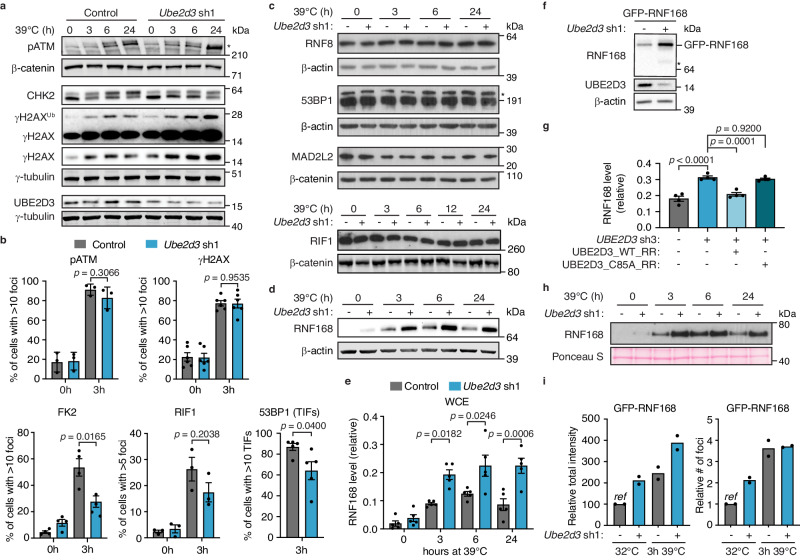
UBE2D3 contributes to DDR-induced chromatin ubiquitination and limits RNF168 protein accumulation and recruitment to uncapped telomeres. **a** Immunoblotting for pATM, γH2AX and CHK2 in TRF2ts MEFs transduced as indicated and upon telomere uncapping at 39 °C. Asterisk indicates ATM phosphorylated at Ser1981. Representative blots from 4 independent experiments. **b** Quantification of pATM, γH2AX, FK2 and RIF1 foci in TRF2ts MEFs, transduced as indicated, at 32 °C (0 h) or upon telomere uncapping for 3 h at 39 °C (*n* = *3* independent experiments for pATM and RIF1, *n* = *6* independent experiments for γH2AX and *n* = *4* independent experiments for FK2; mean ± SEM), and quantification of 53BP1 foci at telomeres (telomere dysfunction-induced foci (TIFs)) in TRF2ts MEFs upon telomere uncapping for 3 h at 39 °C (*n* = *5* independent experiments; mean ± SEM). Statistical significance was calculated using the two-tailed Student’s *t*-test. **c** Immunoblotting for RNF8, 53BP1, RIF1 and MAD2L2 in TRF2ts MEFs subjected to telomere uncapping at 39 °C. Representative blots from 4 independent experiments (RNF8 and 53BP1) or 2 independent experiments (MAD2L2 and RIF1). The asterisk indicates 53BP1; below is a non-specific band. **d** RNF168 levels in TRF2ts MEFs upon telomere uncapping. Representative blots from 5 independent experiments. **e** Quantification of RNF168 levels in whole cell extracts (WCE) (*n* = *5* independent experiments; mean ± SEM; one-way analysis of variance (ANOVA) with Tukey’s multiple comparisons test). **f** Immunoblots for GFP-RNF168 in control and UBE2D3-depleted TRF2ts MEFs. Asterisk indicates endogenous RNF168. Representative blots from 4 independent experiments (quantifications in Supplementary Fig. 5c). **g** Quantification of RNF168 protein levels in HEK 293T cells with complementation of *UBE2D3* depletion by RNAi-resistant (RR) wild-type UBE2D3 (pCDH_UBE2D3_WT_RR) or UBE2D3 C85A (pCDH_UBE2D3_C85A_RR) (*n* = *4* independent experiments; mean ± SEM; one-way analysis of variance (ANOVA) with Tukey’s multiple comparisons test). Representative immunoblots in Supplementary Fig. 5d. **h** Immunoblotting of chromatin fractions to assess RNF168 recruitment to chromatin upon telomere uncapping at 39 °C. Representative blots from 3 independent experiments. **i** Quantification of GFP-RNF168 foci in TRF2ts MEFs at 32 °C and upon 3 h of telomere uncapping at 39 °C. *Ref* reference. Source data are provided as a Source Data file.

To address how UBE2D3 activity promotes NHEJ at telomeres we next asked whether UBE2D3 affects the detection of uncapped telomeres by the DDR machinery. Following telomere uncapping, UBE2D3-depleted TRF2ts cells showed efficient induction of phosphorylated ATM (pATM) and γH2AX on immunoblots, unperturbed accumulation of pATM and γH2AX into DNA damage foci and only marginally affected localisation of pATM to telomeres into telomere dysfunction induced foci (TIFs). This indicates that UBE2D3 does not considerably affect DDR activation at uncapped telomeres (Fig. 3a, b and Supplementary Fig. 4a, b, d, e). However, at 3 h of telomere uncapping UBE2D3-depleted cells accumulated significantly fewer subnuclear foci that are detected with an FK2 antibody following pre-extraction of the cells prior to fixation and staining (Fig. 3b and Supplementary Fig. 4a, b). These foci reflect ubiquitin conjugation at sites of DNA damage or uncapped telomeres, that is indicative of DDR-induced activities of the key ubiquitin E3 enzymes RNF8 and RNF168, involved in recruiting NHEJ promoting factors^7^. This perturbed ubiquitin-dependent DDR signalling in UBE2D3-depleted cells was accompanied by a moderate decrease in 53BP1 foci, only statistically significant when assessed as TIFs, slightly reduced foci formation by its interactor RIF1 (not statistically significant over 3 replicates) and impaired Chk2 phosphorylation (Fig. 3a, b and Supplementary Fig. 4a, c, e, f).

As 53BP1 and RIF1 are known to promote NHEJ, their decreased accumulation could contribute to the impaired NHEJ observed in UBE2D3-depleted cells^3,5,21–28^. However, while this suggests that UBE2D3 promotes NHEJ by contributing to DDR-induced ubiquitination events and 53BP1 recruitment, the relatively modest reduction in 53BP1 accrual seems unlikely to fully account for the substantial NHEJ defect of UBE2D3-depleted cells. Hence, we addressed additional possible roles of UBE2D3 in NHEJ.

### UBE2D3 suppresses RNF168 hyperaccumulation

Besides a regulatory role, UBE2D3-mediated ubiquitination might promote NHEJ by affecting the levels of specific proteins via ubiquitin-mediated proteasomal degradation. Interestingly, while RNF8, 53BP1, RIF1 and MAD2L2 protein levels were unchanged, the levels of RNF168, important for 53BP1 recruitment upon DDR activation^8,9^, were significantly increased in UBE2D3-depleted cells, both before and during TRF2ts inactivation (Fig. 3c–e). This effect was observed in multiple mouse and human cell lines, with independent shRNAs, in the absence and presence of DNA damage induced by irradiation or CRE-mediated deletion of TRF2 and for both endogenous and exogenously expressed RNF168 (Fig. 3d–f and Supplementary Fig. 5a–c). Moreover, the increase in RNF168 upon depletion of UBE2D3 was abrogated by complementation with exogenous shRNA-resistant wild-type UBE2D3, but not catalytically inactive UBE2D3-C85A (Fig. 3g and Supplementary Fig. 5d, e). The latter indicates that the E2 enzyme activity of UBE2D3 is involved in regulating RNF168 protein levels. Concomitant with increased RNF168 protein levels, the association of endogenous RNF168 to chromatin upon telomere uncapping also increased in UBE2D3 depleted cells (Fig. 3h and Supplementary Fig. 5f). To further address whether this involves increased accumulation of RNF168 at uncapped telomeres, we expressed GFP-tagged RNF168 in cells, induced telomere uncapping and assessed the localisation of GFP-RNF168 to sites of DNA damage by immunofluorescence detection of GFP and γH2AX. Indeed, the total intensity of GFP-RNF168 foci in cells subjected to telomere uncapping was increased in UBE2D3-depleted cells, while the average number of GFP-RNF168 foci was not affected (Fig. 3i and Supplementary Fig. 5g, h). Thus, impaired UBE2D3 activity causes both increased levels of total RNF168 protein and hyperaccumulation of RNF168 to sites of DNA damage. This is in line with previous work showing that when RNF168 levels are not tightly controlled and rise to supra-physiological levels, RNF168 spreads over chromatin surrounding DNA breaks^29^.

**Fig. 4:**
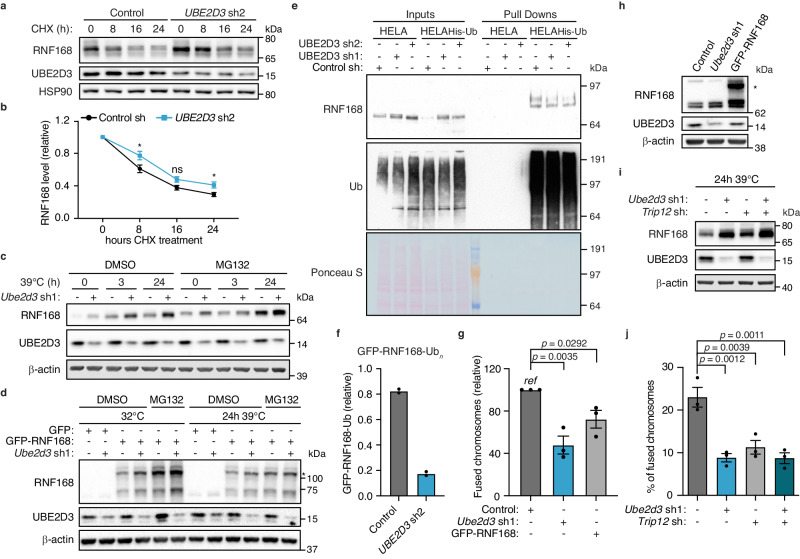
UBE2D3 prevents RNF168 hyperaccumulation through ubiquitination and degradation of RNF168 and thereby facilitates telomere NHEJ. **a** Immunoblots to evaluate RNF168 protein stability in control and *Ube2d3* shRNA2 transduced cells, cultured in the presence of 50 μg/ml cycloheximide (CHX). Representative blots from 5 independent experiments. **b** Quantification of cycloheximide experiments as in a (*n* = *5* independent experiments; mean ± SEM; two-tailed Student’s *t*-test). Significance is shown for Control vs. *UBE2D3* sh2: *p = 0.0488 (8 h), ns *p* = 0.0524 (16 h), *p = 0.0307 (24 h). **c** Immunoblots for RNF168 in control and UBE2D3-depleted TRF2ts cells at 32 °C or upon telomere uncapping and treated for 4 h with 10 μM MG132. Representative blots of two independent experiments. **d** Immunoblots for GFP-RNF168 in control and UBE2D3-depleted TRF2ts cells at 32 °C or upon telomere uncapping and treated for 4 h with 10 μM MG132. Representative blots of two independent experiments. The asterisk annotates the band for GFP-RNF168. **e** HeLa cells with or without expression of His-Ubiquitin were transduced with control or two independent lentiviral shRNAs targeting *UBE2D3*. His-ubiquitin conjugates were purified and analysed by immunoblotting. Representative of 3 independent experiments. See Supplementary Fig. 6b for UBE2D3 levels in input samples. **f** Quantification of polyubiquitinated GFP-RNF168 (Ub^n^) immunoprecipitated from control or UBE2D3-depleted 293T cells, transfected with HA-Ubiquitin and GFP or GFP-RNF168 (*n* = *2* independent experiments). The eluted poly-HA-Ub signal was corrected over the eluted GFP-RNF168 signal. Corresponding blots in Supplementary Fig. 6e, f. **g** Quantification of chromosome fusions in control, *Ube2d3* shRNA1 or GFP-RNF168 transduced TRF2ts cells upon 24 h of telomere uncapping (*n* = *3* independent experiments; mean ± SEM; two-tailed Student’s *t*-test). **h** Immunoblotting for GFP-RNF168 expression and *Ube2d3* depletion in TRF2ts cells used in (**g**). Asterisk indicates GFP-RNF168. **i** Immunoblotting for RNF168 in TRF2ts cells transduced as indicated upon 24 h of telomere uncapping at 39 °C. Representative blots from 3 independent experiments. **j** Chromosome fusions in TRF2ts MEFs transduced with indicated shRNAs upon 24 h of telomere uncapping (*n* = *3* independent experiments; mean ± SEM; one-way analysis of variance (ANOVA) with Tukey’s multiple comparisons test). *Ref* reference. Source data are provided as a Source Data file.

Importantly, no effect was observed on *Rnf168* mRNA levels when UBE2D3 was depleted, indicating that UBE2D3 affects RNF168 solely at its protein level (Supplementary Fig. 6a). Indeed, incubation of cells with the translation inhibitor cycloheximide (CHX) confirmed slower turnover of RNF168 protein in the absence of UBE2D3 (Fig. 4a, b). Furthermore, treatment of control cells with the proteasome inhibitor MG132 resulted in an increase in endogenous and exogenous RNF168 levels, comparable to or surpassing those observed in cells depleted for UBE2D3, in line with UBE2D3 potentially affecting proteasomal degradation of RNF168 (Fig. 4c, d). Indeed, purification of the ubiquitin-proteome from control and UBE2D3-depleted HeLa cells expressing His-tagged ubiquitin, revealed reduced poly-ubiquitination of endogenous RNF168 in UBE2D3-depleted cells (Fig. 4e and Supplementary Fig. 6b–d). Taken together, this suggests that UBE2D3 promotes RNF168 ubiquitination that targets RNF168 for proteasomal degradation. In line with this and with the observed increase in exogenous GFP-RNF168 levels with UBE2D3 depletion, ubiquitination of exogenous GFP-RNF168 was also decreased in UBE2D3-depleted 293T cells (Fig. 4f and Supplementary Fig. 6e, f).

To assess if the aberrant accumulation of RNF168 in UBE2D3-depleted cells contributes to the NHEJ defect of these cells, we overexpressed epitope-tagged RNF168 and assessed telomere fusion efficiency. Indeed, overexpression of GFP-RNF168 resulted in significantly reduced telomere fusion, indicating that aberrantly high RNF168 protein levels interfere with efficient NHEJ (Fig. 4g, h). To independently address this, we made use of an earlier reported finding that the nuclear pool of RNF168 is tightly controlled by the ubiquitin E3 enzymes TRIP12 and UBR5 that ubiquitinate RNF168 and target it for proteasomal degradation^29^. We depleted TRIP12 in TRF2ts cells to increase endogenous RNF168 in the nucleus (Fig. 4i and Supplementary Fig. 6g, h), and assessed the effect on telomere NHEJ. We observed a clear reduction in telomere fusions when TRIP12-depleted cells were exposed to telomere uncapping, similar to cells depleted for UBE2D3 (Fig. 4j and Supplementary Fig. 6h). Moreover, combined depletion of UBE2D3 and TRIP12 did not reduce telomere fusion further (Fig. 4j), indicating that UBE2D3 and TRIP12 promote NHEJ through the same mechanism and supporting that this is mediated via control of RNF168 accumulation.

### UBE2D3 restricts PP2A-mediated KAP1 dephosphorylation

**Fig. 5:**
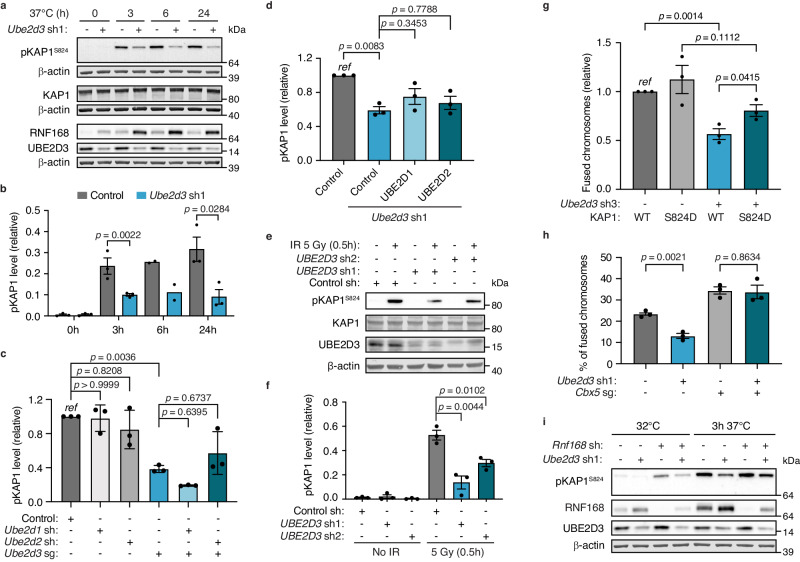
UBE2D3 promotes KAP1-S824 phosphorylation via regulation of RNF168 stability, thereby facilitating NHEJ at telomeres. **a** Immunoblotting for pKAP1 (S824), KAP1 and RNF168 in control and UBE2D3-depleted TRF2ts MEFs upon telomere uncapping at 37 °C. Representative blots from 3 independent experiments. **b** Quantification of pKAP1 levels in TRF2ts MEFs transduced as indicated. The mean ± SEM from *n* = *3* independent experiments is shown, except for the 6 h timepoint that is *n* = *2*. Statistical significance was calculated using one-way analysis of variance (ANOVA) with Tukey’s multiple comparisons test. **c** Quantification of pKAP1 levels in TRF2ts MEFs transduced with *Ube2d1* shRNA, *Ube2d2* shRNA and/or *Ube2d3* sgRNA upon 3 h of telomere uncapping at 37 °C (*n* = *3* independent experiments; mean ± SEM; one-way analysis of variance (ANOVA) with Tukey’s multiple comparisons test). **d** Quantification of pKAP1 levels in TRF2ts MEFs transduced with *Ube2d3* sh1 and complemented with exogenously expressed control, 3xFLAG-UBE2D1 or 3x-FLAG-UBE2D2 constructs (*n* = *3* independent experiments; mean ± SEM; one-way analysis of variance (ANOVA) with Tukey’s multiple comparisons test). **e** Immunoblotting for pKAP1 (S824) in HeLa cells transduced with control or *UBE2D3* shRNAs at 30 min post IR (5 Gy). Representative blots from 3 independent experiments. **f** Quantification of pKAP1 levels in HeLa cells upon IR (representative immunoblots in (**e**)). The mean ± SEM from 3 independent experiments is shown. Statistical significance was calculated using the two-tailed Student’s *t*-test. **g** Chromosome fusions in TRF2ts MEFs transduced with control or *Ube2d3* shRNA3, complemented with exogenously expressed KAP1 WT or KAP1 S824D mutant, upon 36 h of telomere uncapping at 37 °C (*n* = *3* independent experiments; mean ± SEM; two-tailed Student’s *t*-test). **h** Chromosome fusions in TRF2ts MEFs transduced with *Ube2d3* sh1 and/or an sgRNA targeting *Cbx5* (encoding the HP1α protein), along with non-targeting control shRNA/sgRNA (-) where appropriate (*n* = *3* independent experiments; mean ± SEM two-tailed Student’s *t*-test). **i** Immunoblotting for pKAP1 (S824) in TRF2ts MEFs transduced with control, *Ube2d3* and/or *Rnf168* shRNAs at 32 °C or after 3 h at 37 °C. Representative blots from 2 independent experiments. *Ref* reference. Source data are provided as a Source Data file.

While investigating how reduced UBE2D3 activity and aberrant RNF168 stabilisation result in NHEJ impairment, we observed a substantial defect in ATM-dependent phosphorylation of KAP1-Serine 824 (S824) in UBE2D3-depleted cells. This was observed both upon telomere deprotection in MEFs and upon DNA damage induced by irradiation of HeLa cells (Fig. 5a, b, e, f and Supplementary Fig. 7a, b). In line with our assessment of cell viability upon TRF2 inactivation (Fig. 1d and Supplementary Fig. 1e–h), we did not detect a significant contribution of UBE2D1 or UBE2D2 to KAP1 phosphorylation upon telomere uncapping. The shRNA-mediated knockdown of UBE2D1 or UBE2D2 on top of sgRNA-mediated depletion of UBE2D3, did not aggravate the KAP1 phosphorylation defect of UBE2D3-depleted cells, only a marginal (statistically nonsignificant) additional effect was seen for UBE2D1 depletion (Fig. 5c and Supplementary Fig. 7c). Also, overexpression of exogenous UBE2D1 or UBE2D2 did not rescue the KAP1 phosphorylation defect of UBE2D3-depleted cells (Fig. 5d and Supplementary Fig. 7d).

As ATM-dependent phosphorylation of the heterochromatin factor KAP1 at S824 is essential for DNA repair at heterochromatin^30–32^, we assessed if impaired KAP1 phosphorylation could explain the defective NHEJ at telomeres in UBE2D3-depleted cells. Indeed, expression of KAP1 with the phosphomimetic S824D mutation was able to restore NHEJ-mediated telomere fusion in UBE2D3-depleted cells (Fig. 5g and Supplementary Fig. 7e), indicating that efficient telomere NHEJ requires KAP1-S824 phosphorylation. Since at heterochromatin the loss of Heterochromatin Protein 1 (HP1), a binding partner of KAP1, was shown to bypass the requirement for ATM in DNA double-strand break (DSB) repair^30^, we performed a rescue experiment by depleting HP1α, using sgRNA targeting the mouse *Cbx5* gene (encoding HP1α). Ablation of HP1α fully restored the NHEJ defect caused by UBE2D3 depletion, indicating that UBE2D3 facilitates telomere NHEJ in a manner depending on HP1 (Fig. 5h and Supplementary Fig. 7f).

Interestingly, also hyperaccumulation of RNF168 upon TRIP12 and UBR5 depletion was previously found to be associated with impaired KAP1-S824 phosphorylation^29^. This suggests that the reduced KAP1-S824 phosphorylation in UBE2D3-depleted cells might relate to the hyperaccumulation of RNF168 in these cells. Indeed, depletion of RNF168 on top of UBE2D3 depletion, partially restored phosphorylation of KAP1 (Fig. 5i and Supplementary Fig. 7g).

**Fig. 6:**
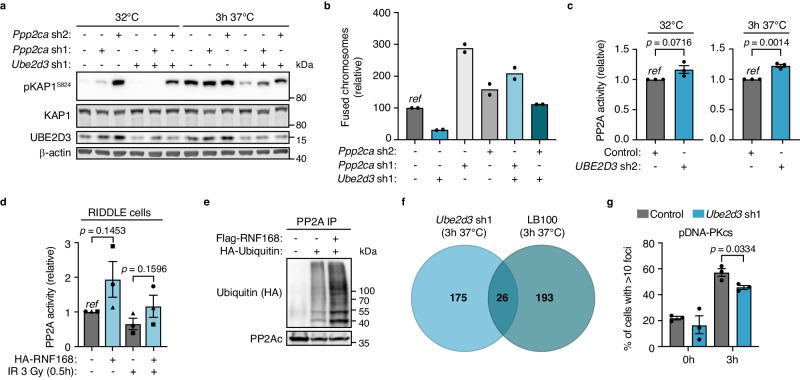
UBE2D3 promotes KAP1 phosphorylation and telomere NHEJ in a PP2A-dependent manner. **a** Immunoblotting for pKAP1 (S824) in TRF2ts MEFs transduced with control, *Ube2d3* and/or two independent *Ppp2ca* shRNAs at 32 °C or after 3 h at 37 °C. Representative blots from 2 independent experiments. **b** Quantification of chromosome fusions in TRF2ts MEFs transduced with control, *Ube2d3* and two independent *Ppp2ca* shRNAs, upon 24 h of telomere uncapping. Two independent experiments are shown. **c** PP2A activity assays with immunoprecipitated PP2A from TRF2ts MEFs transduced as indicated and cultured at 32 °C or for 3 h at 37 °C to induce telomere uncapping (*n* = *3* independent experiments; mean ± SEM; two-tailed Student’s *t*-test). Immunoblots of input and immunoprecipitates are shown in Supplementary Fig. 8e. **d** PP2A phosphatase activity assay with immunoprecipitated PP2A-alpha (PP2Ac) from RNF168 mutant human cells (RIDDLE) with and without expression of ectopic HA-RNF168. Corrected for the amount of immunoprecipitated PP2A-alpha. Cells were untreated or harvested 30 min after irradiation with 3 Gy (*n* = *3* independent experiments; mean ± SEM; two-tailed Student’s *t*-test). The different symbols (dot, square and triangle) represent 3 biologically independent experiments. Immunoblots of input and immunoprecipitates are shown in Supplementary Fig. 8f. **e** Assessment of in vivo PP2A ubiquitination by immunoprecipitation of endogenous PP2A catalytic C subunit from HEK 293T cell lysates transfected with the indicated plasmids. See Supplementary Fig. 8i for input samples. Representative blots from 3 independent experiments. **f** Venn diagram showing the overlap in significantly altered phospho-substrates in 3 independent replicate phosphoproteomics analyses of UBE2D3-depleted (*Ube2d3* sh1) or PP2A inhibitor-treated (5 μM LB100, 15 h) TRF2ts MEFs upon 3 h of telomere uncapping at 37 °C. Numbers indicate the phospho-substrates that were significantly reduced in UBE2D3-depleted cells or significantly increased in LB100-treated cells. *Ube2d3* sh1 and LB100 were compared to control (shScramble) TRF2ts MEFs. **g** Quantification of pDNA-PKcs (S2056) foci in TRF2ts MEFs, transduced as indicated, at 32 °C (0 h) or upon telomere uncapping for 3 h at 39 °C (*n* = *3* independent experiments; mean ± SEM; two-tailed Student’s *t*-test). *Ref* reference. Source data are provided as a Source Data file.

Next, we aimed to understand how impaired UBE2D3 activity and aberrantly high RNF168 levels could result in compromised KAP1 phosphorylation. As ATM kinase activity was unperturbed in UBE2D3-depleted cells, as evidenced by efficient ATM and H2AX phosphorylation (Fig. 3a, b and Supplementary Fig. 4a, b) and therefore unlikely responsible for the reduced pKAP1-S824, we turned our attention to protein phosphatases that potentially counteract KAP1 phosphorylation. Analysis of diGly-proteomics that we performed to identify changes in protein ubiquitination following UBE2D3 depletion^33^, suggested increased ubiquitination of lysine 21 of PP2A-alpha, a catalytic C subunit of PP2A, which is one of the main serine/threonine-protein phosphatases^34^. Although the relevance of PP2A-alpha lysine 21 ubiquitination for PP2A activity is unknown, this points at PP2A-alpha (encoded by *Ppp2ca)* as a potential indirect substrate of UBE2D3 that may contribute to deregulation of KAP1 phosphorylation in UBE2D3-depleted cells. In particular, as PP2A is one of the phosphatases known to remove phosphorylation on S824 of KAP1^35^. To address if the decreased KAP1 phosphorylation and impaired NHEJ in UBE2D3-depleted cells are potentially involving PP2A-alpha, we co-depleted PP2A-alpha with UBE2D3 and examined pKAP1-S824 levels and NHEJ efficiency. Indeed, pKAP1-S824 levels in UBE2D3-depleted cells were partially restored upon PP2A-alpha depletion with *Ppp2ca* shRNAs, suggesting that the negative impact of UBE2D3 depletion on KAP1-S824 phosphorylation is at least partially attributable to PP2A activity (Fig. 6a and Supplementary Fig. 8a–c). Moreover, PP2A-alpha depletion increased NHEJ at deprotected telomeres, while UBE2D3-depleted cells, normally impaired in NHEJ, remained proficient in telomere NHEJ upon co-depletion of PP2A-alpha with *Ppp2ca* shRNAs (Fig. 6b and Supplementary Fig. 8a). In line with PP2A being affected in UBE2D3-depleted cells via a regulatory effect on the PP2A-alpha catalytic C subunit, UBE2D3 depletion was accompanied by a modest increase in overall cellular PP2A enzyme activity (measured against an in vitro provided peptide substrate), with no changes in the overall PP2A-alpha protein levels (Fig. 6c and Supplementary Fig. 8d, e). Of note, elevated PP2A activity may also explain the reduced ATM-dependent CHK2 phosphorylation observed in UBE2D3-depleted cells despite seemingly unperturbed ATM activity, as CHK2 is a known target of PP2A (Fig. 3a and Supplementary Fig. 4f)^36^. Moreover, we detected increased PP2A activity, as well as impaired KAP1-S824 phosphorylation, in RIDDLE syndrome patient cells lacking functional RNF168^8^, following their reconstitution with exogenous RNF168. Although elevated PP2A activity was detected in each of 3 independent experiments, the extent of this increase, unlike that of pKAP1-S824, did not hold statistical significance over 3 independent replicates, due to large variation amongst the measured PP2A activity, with one of three experiments displaying considerable milder differences, both on PP2A activity and KAP1 phosphorylation (Fig. 6d and Supplementary Fig. 8f, g). Nevertheless, the data are in support of a stimulatory effect of RNF168 on PP2A activity that could contribute to enhanced KAP1 dephosphorylation and provide an explanation for the reduced pKAP-S824 seen upon RNF168 hyperaccumulation, such as in UBE2D3-depleted cells or upon reconstituting RIDDLE cells with ectopic RNF168. Furthermore, overexpression of RNF168 enhanced ubiquitination of PP2A-alpha in vivo (Fig. 6e and Supplementary Fig. 8i), further suggesting that RNF168 might be involved in regulating PP2A activity via the ubiquitination of PP2A-alpha.

Given the modest or variable effects measured in PP2A activity assays (using an in vitro substrate), we further assessed the impact of UBE2D3 on phospho-substrates in vivo, by performing phosphoproteomic analysis of control, UBE2D3-depleted and PP2A inhibitor (LB100)-treated TRF2ts MEFs (Fig. 6f and Supplementary Fig. 9a–d). Of the phospho-substrates that were significantly increased upon LB100 treatment, 11.8% (26 out of 219), were also decreased in UBE2D3-depleted cells, supporting that UBE2D3 affects a portion of PP2A substrates. UBE2D3 depletion also changed phospho-targets that were not changed in LB100-treated cells. This may reflect differences in treatment duration between inhibitor and shRNA mediated inhibition or indicate that UBE2D3, directly or indirectly, affects additional phosphatases or kinases. Interestingly, several DNA repair proteins, including XRCC5, 53BP1, LIG3 and RECQL5, were underphosphorylated upon telomere deprotection in UBE2D3-depleted cells (Supplementary Fig. 9a–d). This points at a potential broader role for UBE2D3 in phospho-regulation of DDR factors upon telomere deprotection. Although the significance of the differential phosphorylation of these factors is unclear at this point, it is possible that these changes contribute to impaired NHEJ in UBE2D3-depleted cells or reflect a consequence thereof.

Finally, we examined the core NHEJ-machinery and assessed the activation and recruitment of DNA-PKcs that is critical for NHEJ^37^, by quantifying subnuclear foci of DNA-PKcs (auto)phosphorylated at Serine 2056 upon telomere uncapping (Fig. 6g and Supplementary Fig. 8h). UBE2D3-depleted cells showed a mild but significant reduction in pDNA-PKcs foci upon telomere uncapping, which could be contributing to the NHEJ defect of these cells and be a downstream consequence of the RNF168 hyperaccumulation, phosphatase-deregulation and KAP1- and HP1-mediated restriction on end-joining in UBE2D3-depleted cells.

**Fig. 7:**
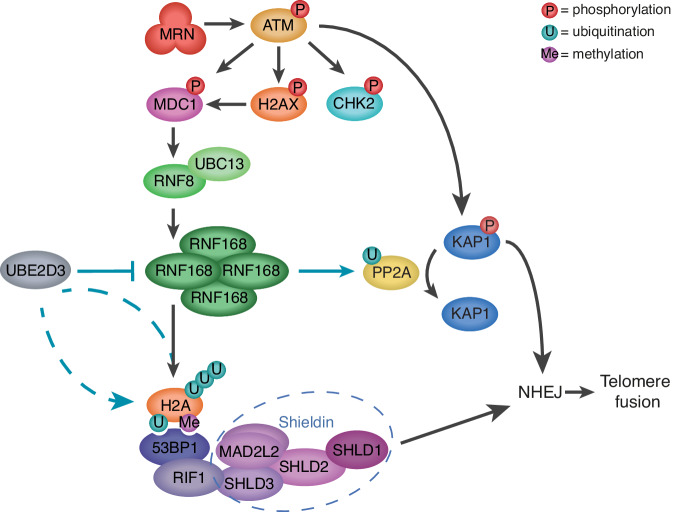
Model for how UBE2D3 facilitates NHEJ by promoting ATM kinase-dependent DDR activities. On the one hand UBE2D3 promotes DDR-induced chromatin ubiquitination and 53BP1 recruitment, that are known to be mediated by the RNF8 and RNF168 E3 ligases following ATM kinase activation. 53BP1 is known to promote NHEJ by recruiting the shieldin complex and promoting chromatin mobility (not depicted). Thus, UBE2D3 appears to support the role of RNF168 in promoting NHEJ via chromatin ubiquitination and 53BP1 recruitment. On the other hand, UBE2D3 promotes the ubiquitination and proteasomal degradation of RNF168, thereby preventing RNF168 hyperaccumulation. UBE2D3-deficiency and RNF168 hyperaccumulation are associated with impaired KAP1-S824 phosphorylation, that appears to be in part caused by enhanced phosphatase activity by PP2A, but may also involve other mechanisms. By limiting RNF168 accumulation, UBE2D3 prevents disproportionate dephosphorylation of KAP1 that counteracts the ability of ATM to promote efficient NHEJ through phosphorylation of S824 of KAP1. Thus, this implies the existence of a negative feed-back circuit within ubiquitin and kinase signalling in the DDR that is constrained by UBE2D3 and consists of RNF168- and phosphatase-mediated restriction of ATM-dependent KAP1 phosphorylation.

Together this work indicates that efficient NHEJ not only relies on UBE2D3- and RNF168-supported recruitment of NHEJ-promoting factors following ATM kinase activation, but also requires UBE2D3-mediated restriction of RNF168 accumulation and (PP2A) phosphatase activity to avoid excessive inactivation of ATM-dependent KAP1 phosphorylation (Fig. 7). Thus, these results reveal the existence of a negative regulatory circuit within ubiquitin and kinase signalling in the DDR that is constrained by UBE2D3.

## Discussion

UBE2D3 has been implicated in multiple cellular processes, including in homologous recombination at DNA DSBs where it acts with RNF138 in promoting CtIP ubiquitination and accrual^38^. Here we identify a role for UBE2D3 as a facilitator of NHEJ-mediated DNA repair, where it acts by promoting ATM kinase-initiated DDR activities through both supporting and restraining different RNF168 activities. On one hand UBE2D3 contributes to ATM-induced chromatin ubiquitination and 53BP1 recruitment that support NHEJ and are known to be promoted by RNF168^8,9^, itself recruited in an ATM-dependent manner. On the other hand, UBE2D3 facilitates ATM-mediated phosphorylation of KAP1, which is essential for efficient NHEJ at telomeres. It achieves this by promoting ubiquitination and subsequent degradation of RNF168, thereby preventing RNF168 hyperaccumulation that is associated with impaired KAP1 phosphorylation. Our results suggest that this process involves the regulation of PP2A phosphatase activity, which counteracts the ATM kinase activity directed towards KAP1.

In previous work we showed that depletion of E3 enzymes RNF8 or RNF168 reduces genomic instability resulting from NHEJ at uncapped telomeres, in line with their established roles in promoting NHEJ at DSBs^5,8,9^. RNF168 is known to govern mono-ubiquitination of histone H2A(X) at lysine 13/15 (K13/15) following DNA damage-induced ATM activation^39^. The H2A(X)K15 histone modification is bound by the ubiquitin-dependent recruitment domain of 53BP1 and is critical for the accumulation of 53BP1 and its downstream factors, such as RIF1 and MAD2L2, to sites of DNA damage^3,21–28,39,40^. In addition, RNF168 removes factors that compete with 53BP1 for binding to dimethylated histone H4K20^41,42^ and catalyses K63-linked ubiquitin-chain formation on 53BP1 to promote its initial DSB recruitment^43^. However, despite increased RNF168 accumulation in UBE2D3-depleted cells, these cells did not show enhanced recruitment of 53BP1, but in fact showed reduced ubiquitin conjugation and 53BP1 accumulation at damaged chromatin. This is intriguing, as enhanced RNF168 accumulation and spreading around sites of DNA damage is expected to result in increased 53BP1 recruitment, as was indeed observed for RNF168 hyperaccumulation following TRIP12/UBR5-loss in experimental settings and human malignancies^29^. The lack of increased RNF168-dependent accumulation of 53BP1 and RIF1, and moreover, a reduction in DNA-damage induced ubiquitin conjugation (FK2) and 53BP1 recruitment in UBE2D3-depleted cells, suggests that RNF168 may need UBE2D3 as an E2 to promote 53BP1 recruitment via ubiquitination of H2A(X)-K13/15 and potentially additional substrates. In fact, it was shown that RNF168 and UBE2D3 can act as an E3/E2 pair to ubiquitinate H2AK13/15 in vitro^39^. However, UBE2D3 is very promiscuous in vitro and whether UBE2D3 is also the E2 involved in vivo has not been confirmed. Our observations here are in support of UBE2D3 also acting with RNF168 in vivo, but this requires further assessment. UBE2D3 may also have other direct or indirect activities that explain why enhanced RNF168 recruitment does not cause increased ubiquitin conjugation and 53BP1 accrual in UBE2D3-deficient cells.

Although the modestly reduced 53BP1 recruitment could partially account for the reduced NHEJ in cells with diminished UBE2D3, the more prominent consequences of UBE2D3 depletion are hyperaccumulation of RNF168 and impaired KAP1-S824 phosphorylation. Our identification of UBE2D3 as a negative regulator of RNF168 accumulation is in line with *UBE2D3* appearing as an uncharacterised hit in a previous screen searching for genes whose knockdown increased the expansion of GFP-RNF168-decorated nuclear bodies^29^. This screen identified TRIP12 and UBR5 as E3 ligases that control accumulation of RNF168, with TRIP12 acting specifically on RNF168, while UBR5 more broadly affects the levels of multiple DDR factors, including RNF8. Our findings show that UBE2D3, like TRIP12, prevents the hyperaccumulation of RNF168 specifically. Additionally, both UBE2D3 and TRIP12 promote NHEJ in an epistatic manner. These observations suggest the possibility that UBE2D3 may act as an E2 together with TRIP12 to ubiquitinate RNF168 and target it for proteasomal degradation. However, we have been unable to conclusively demonstrate that TRIP12 requires UBE2D3 for this activity in vivo, thus leaving open the possibility that UBE2D3 is directly or indirectly involved in RNF168 ubiquitination and degradation through other mechanisms.

Both UBE2D3-deficiency and RNF168 hyperaccumulation are associated with reduced KAP1-S824 phosphorylation. Our data indicate that this is the result of inappropriate dephosphorylation of pKAP1-S824 by phosphatase activity, rather than a defect in KAP1-S824 phosphorylation by ATM. Reduced pKAP1-S824 in UBE2D3-deficient cells was dependent on RNF168, suggesting that UBE2D3-deficiency, via aberrant stabilisation of RNF168, results in elevated dephosphorylation of pKAP1-S824. Since expression of KAP1 with the phosphomimetic S824D mutation rescued telomeric NHEJ in UBE2D3-depleted cells and depletion of the PP2A-alpha catalytic C subunit of PP2A also rescued both KAP1-S824 phosphorylation and telomeric NHEJ in these cells, inappropriate dephosphorylation of pKAP1-S824 appears to underlie the NHEJ-defect in UBE2D3-depleted cells. Our data point at the involvement of dephosphorylation by PP2A but do not exclude the involvement of other modes of KAP1 phospho-regulation, as PP2A inhibition may have pleiotropic effects.

The restoration of NHEJ-mediated telomere fusion in UBE2D3-depleted cells by expression of ectopic phosphomimetic KAP1^S824D^ or ablation of HP1α phenotypically resembles the need for chromatin relaxation at heterochromatin to repair DSBs^30^. This suggests that UBE2D3-dependent KAP1 phosphorylation promotes telomere NHEJ by modification of the telomeric chromatin state and may serve to alleviate a certain telomere-specific hindrance on DNA repair imposed by the heterochromatin factors KAP1 and HP1. However, the nature of this hindrance at telomeres requires further investigation in future work, as previous reports indicated the lack of and no need for, chromatin relaxation/decompaction at deprotected telomeres^44,45^.

While diGly proteomics analyses hint at the possible involvement of PP2A-alpha lysine 21 ubiquitination^33^, how UBE2D3 and RNF168 indirectly or directly impact PP2A activity towards KAP1 requires further investigation. This is warranted since members of the PP2A family of serine/threonine phosphatases are important tumour suppressor genes, yet the regulation of PP2A activity and specificity is complex and far from being fully understood. This is complicated by the fact that rather than representing a single enzymatic activity, the PP2A holoenzyme comprises multiple distinct heterotrimeric enzymes, with each holoenzyme consisting of a complex of different subunits with distinct substrate and subcellular specificities^46^. Although lysine 21 is one of the residues known to be ubiquitinated at the N-terminus of PP2A-alpha, the relevance of this ubiquitination is not known^47^. Hence, it is at this point unclear if and how it might be involved in enhancing phosphatase activity towards KAP1. Regulation of PP2A-alpha protein stability seems not to be involved, as PP2A-alpha levels were unchanged in UBE2D3-deficient cells or upon exogenous expression of RNF168 in RIDDLE cells, pointing at an alternative mode of PP2A regulation that is an interesting direction in future research.

Our results that suggest a regulatory role for RNF168 on PP2A, whether direct or indirect, are further supported by recent work on inhibition of the deubiquitinating enzyme (DUB) USP7. In this work, USP7 inhibition was found to reduce PP2A activity and increase KAP1-S824 phosphorylation^48^. Although this was ascribed to an ability of USP7 to interact with PP2A and affect its localisation, it is important to note in the context of our work that USP7 was previously also reported to stabilise RNF168^48^. Moreover, we noticed in other previous work that inhibition of USP7, besides being associated with increased ubiquitination of the PPP2R1A subunit of PP2A, is associated with decreased ubiquitination of PPP2CA (PP2A-alpha) at lysine 21^49^. The latter is likely an indirect effect, since inhibition of a DUB is expected to result in increased ubiquitination of its direct targets. Together, these independent findings on USP7, along with our results reported here, suggest that RNF168 stabilisation by USP7 might contribute to the effects of USP7 on PP2A activity and pKAP1-S824 and that RNF168 might stimulate PP2A activity via ubiquitination of the PP2A-alpha catalytic C subunit of PP2A. However, whether PP2A is a direct target of RNF168 and how it would affect PP2A activity, remains to be addressed in future work.

Together, this work provides insights into the regulation of DNA repair through the concerted action of ubiquitin machinery enzymes, phosphatase activity and ATM kinase activity, that ensures appropriate DDR signalling responses to promote efficient DNA repair. We uncover roles of UBE2D3, RNF168 and PP2A in genome stability maintenance, in which UBE2D3, besides supporting RNF168-mediated chromatin ubiquitination and 53BP1 accrual, acts to prohibit RNF168 hyperaccumulation and thereby prevents aberrant dephosphorylation of KAP1. This would otherwise impair NHEJ at genomic regions requiring KAP1 phosphorylation for effective DNA repair. Our work further supports the earlier notion that the nuclear pool of RNF168 needs to be tightly regulated for appropriate cellular responses to DNA damage. However, we now include UBE2D3 as an important factor in this regulation and point at a previously unrecognised way of how supra-physiological levels of RNF168 may alter DNA repair efficiency, separately from 53BP1 recruitment, i.e. via control of KAP1 dephosphorylation. Hyperaccumulation of RNF168 in cancer has been proposed to be a modifier of chemosensitivity by accelerating repair of clastogen-induced DSBs and increasing mutagenic repair by NHEJ in the context of BRCA1-deficiency, through enhanced 53BP1 accrual^29,50^. Our work here suggests alternative consequences of RNF168 hyperaccumulation, namely altered phosphatase activity and impaired DNA repair in genomic regions that rely on KAP1 phosphorylation for efficient repair, such as heterochromatin and including, as we show here, telomeres.

## Methods

### Screen

*Trf2* ^*-/-*^*;p53* ^*-/-*^;TRF2ts MEFs (TRF2ts MEFs) were infected with retroviral pRetrosuper shRNA pools of the Netherlands Cancer Institute (NKI) mouse shRNA library that targets ~15,000 genes with two different shRNA constructs per gene^11^. As a control, cells were infected with virus generated from an empty pRetrosuper retroviral vector. Cells were plated at 150,000 cells per 15 cm dish and, after adherence at 32 °C, cultured for 12 days at 39 °C, followed by culturing at 32 °C. Outgrowth of cell colonies was monitored for multiple weeks. Several hundred single colonies of library-infected cells were picked, expanded and processed for genomic DNA isolation. ShRNA inserts were recovered by PCR (long-template PCR kit, Roche) using the following primers: Fw: 5’-CTTGAACCTCCTCGTTCGACC-3’ and Rv: 5’-CTAAAGCGCATGCTCCAGACTG-3’. Purified PCR products were digested with EcoRI and XhoI, and fragments containing the H1 promoter and the shRNA hairpin sequence were re-cloned into the pRetrosuper backbone for validation and sequencing. Target sequences were identified using NCBI’s Basic Logic Alignment Search Tool (BLAST).

### Cell culture, growth assays and flow cytometry

*Trf2* ^*-/-*^*;p53* ^*-/-*^;TRF2ts MEFs (TRF2ts MEFs) were generated from *Trf2* ^*flox/-*^;*p53* ^*-/-*^ MEFs as described before^5^. *Trf2* ^*flox/-*^*;p53*^*-/-*^ MEFs were obtained from T. de Lange, SV40-immortalised WT MEFs were obtained from R. Chapman, *Trf2* ^fl/fl^;*Rosa26-CreERT2* MEFs were obtained from E. Lazzerini Denchi, RIDDLE cells^8^ with and without RNF168 reconstitution were obtained from F. Mattiroli, doxycycline-inducible shTRF2 HeLa cells^51^ were obtained from J. Lingner. Phoenix producer cells, HEK 293T, U2OS and HeLa cells were obtained from ATCC. All cells were grown in DMEM with 100 U penicillin, 0.1 mg ml^−1^ streptomycin, 2 mM L-glutamine and 10% FBS. TRF2ts MEFs were maintained at the permissive temperature of 32 °C and only grown at 37 °C or 39 °C to induce telomere uncapping through inactivation of TRF2. All other cells were grown at 37 °C.

For survival assays, TRF2ts MEFs were plated at 150,000 cells per 15 cm dish or 45,000 per 10 cm dish, allowed to adhere and either kept at 32 °C or placed at 39 °C. To evaluate potential toxicity or growth rate differences, plates were fixed with 4% formaldehyde and stained with 0.1% crystal violet after growth for 1 week at 32 °C. To address survival upon prolonged telomere uncapping, plates were fixed and stained after 12 days of growth at 39 °C and recovery for 2–4 weeks at 32 °C. Quantifications of survival assays reflect the relative survival after growth for 12 days at 39 °C and 2 weeks recovery at 32 °C, corrected for plating efficiency and growth at 32 °C and assessed by crystal violet extraction, as described below.

To address cell survival under telomere uncapping conditions in short-term growth assays, TRF2ts MEFs (clone B17) were plated in quadruplicate at 5000 cells per well in 12-well plates, allowed to adhere overnight at 32 °C and then placed for 12–14 days at 39 °C. During this time of culturing at the non-permissive temperature, plates were fixed every 2–4 days with 4% formaldehyde and stained with 0.1% crystal violet. Crystal violet was extracted from the plates using 10% acetic acid and its absorbance at 595 nm was quantified using a Tecan microplate reader. Quantifications were corrected for plating efficiency. Alternatively, for growth assay as shown in Fig. 1c, TRF2ts MEFs (clone C15) were plated in triplicate at 5000 cells per well in 6-well plates, allowed to adhere overnight at 32 °C and then placed for 9 days at 39 °C. Plates were fixed and stained with crystal violet on day 0, 1, 3, 6, 9, as described above and analysed for cell density using ImageJ.

Cell cycle distribution of TRF2ts MEFs with or without *Ube2d3* knockdown was determined by flow cytometry analysis of propidium iodide staining and BrdU incorporation, acquired on a BD Fortessa using FACSDiva software (BD Biosciences) and analysed with FlowJo (TreeStar) software.

Analysis of aneuploidy (>4 *N*) was based on DNA-content analysis by flow cytometry on a BD FACSCalibur of TRF2ts cells grown at 32 °C or 39 °C for 48 h and then stained with propidium iodide. Fold increase in aneuploidy was calculated by comparing the fraction of cells with >4 N DNA content upon 48 h of telomere uncapping at 39 °C with no uncapping at 32 °C (39 °C/32 °C) for each experimental condition.

For intracellular FACS staining for DNA damage markers, cells were seeded at 50% confluency the day before the experiment. After treatment, cells were trypsinised and resuspended in ice-cold PBS with 10% FCS and centrifuged at 280 *g* for 5 min at 4 °C. Cell pellets were resuspended in 0.5 ml ice-cold PBS and fixed by adding dropwise 4.5 ml of ice-cold 100% methanol, while vortexing. Cells were incubated overnight at −20 °C before start of the staining. After adding 5 ml PBS, fixed cells were centrifuged (438 *g*, 4 min at RT), resuspended in 500 μl PBS/0,5% BSA (PBA), transferred to a 96-wells plate and centrifuged (438 *g*, 5 min at RT). Cells were permeabilised for 15 min on ice in PBA with 0,1% Triton. Next, cells were washed 3x with PBA and incubated with pKAP1 antibody (1:250; Bethyl A300-767A) for 3 h at RT, while shaking. Subsequently, cells were washed 3x with PBA and incubated with Alexa 647 (1:5000, Invitrogen A21246) for 1 h at RT, while shaking. Cells were washed 3x in PBA and finally resuspended in DAPI (1:1000 dilution of 5 mg/ml DAPI in PBA) and analysed by flow cytometry on a BD Fortessa FACS machine. FACS data were analysed by FlowJo software.

### Retroviral and lentiviral transductions

For retroviral infection, ecotropic phoenix producer cells (ATCC) were seeded at 2.5 × 10^6^ cells per 10 cm dish and transfected using CaPO_4_ precipitation of 20 μg of retroviral vector DNA. Medium was refreshed at 16 and 24 h after adding the DNA/CaPO_4_ precipitate, and viral supernatants were collected at ~48, 62 and 72 h post-transfection and filtered through a 0.22 μM syringe filter. Viral supernatants were either frozen on dry ice and stored at −80 °C until use, or used immediately. For infection, cells were overlaid with viral supernatant, supplemented with 4 μg ml^−1^ polybrene. Cells were infected at least twice to achieve 100% infection efficiency, which was confirmed by acquired resistance to selection drugs (2–4 μg ml^−1^ puromycin or 5 μg ml^−1^ blasticidin (Invitrogen)). Cells were transduced with pRetrosuper-puro retroviruses encoding shRNAs targeting mouse and human *Ube2d3* shRNA1: 5’-TGGGTTTGGATCACATATC-3’, mouse *Ube2d3* shRNA2: 5’-GTGGTCTCCTGCTTTAACT-3’, mouse *Nbs1* shRNA1: 5’-TTTGACTCAAACTGGTTAC-3’, mouse *Lig4* shRNA1: 5’-GGATCAGAGACGAGTTACT-3’, mouse and human *TRF2* shRNA2: 5’-GAACAGCTGTGATGATTAA-3’, human *UBE2D3* shRNA2: 5’-CCAGAGATTGCACGGATCTAT-3’. The use of TRF2 shRNA2 has been described before^5,13^.

For complementation experiments, human UBE2D3 cDNA was first cloned into the Gateway entry vector pENTR and then shuttled into an LZRS-ires-GFP backbone using Gateway technology (Invitrogen). In addition to the wild-type (WT) construct, a UBE2D3 C85A mutant construct was created using QuickChange Site-Directed mutagenesis (Stratagene). In both the WT and C85 mutant construct, the target sequence of *Ube2d3* shRNA1 was changed at four positions with silent mutations to create RNAi-resistant constructs. UBE2D3 WT and C85A mutant RNAi-resistant cDNAs were also cloned into a pCDH-puro backbone using conventional cloning. Expression constructs of UBE2D1 and UBE2D2 were obtained by PCR amplifying human UBE2D1 and UBE2D2 cDNA with the addition of an N-terminal 3xFLAG epitope tag. The amplicons obtained were then subcloned into a pCDH-BLAST backbone using conventional cloning.

For localisation and overexpression experiments, human *RNF168* cDNA was Gateway-cloned (Invitrogen) into a pMSCV-retroviral vector containing a coding sequence for an amino-terminal GFP tag.

For lentiviral infection, HEK 293T cells (ATCC) were transfected using CaPO_4_ precipitation of 10 μg pLKO-puro lentiviral vector DNA obtained from Mission shRNA library clones (Sigma-Aldrich). Target cells were incubated for 6 h with viral supernatant that was supplemented with 4 μg ml^−1^ polybrene. The following lentiviral shRNAs were used: mouse *Ube2d1* shRNA TRCN0000040893: 5’-GCACACTGTATTTCTCAGTAT-3’, mouse *Ube2d2* shRNA TRCN0000037318: 5’-CCTGTTGGAGATGATATGTTT-3’, mouse *Ube2d3* shRNA #3 TRCN0000039469: 5’-ACAACAGAATATCTCGGGAAT-3’, mouse *Rnf168* TRCN0000040875: 5’-GCAGGACAGATTGTTAGCATT-3’, mouse *Trip12* shRNA TRCN0000039425: 5’-CGGGCTGTGAAGTTCTTGTTT-3’, mouse *Ppp2ca* shRNA1 TRCN0000081363: 5’-GCGACATTGTTGGTCAAGAAT-3’, mouse *Ppp2ca* shRNA2 TRCN0000081365: 5’-CGACGAGTGTTTAAGGAAATA-3’, human *UBE2D3* shRNA1 TRCN0000038790: 5’-GCCTGCTTTAACAATTTCTAA-3’, human and mouse *UBE2D3* shRNA2 TRCN0000038792: 5’-CCAGAGATTGCACGGATCTAT-3’, human *UBE2D3* shRNA3 5’-TGGGTTTGGATCACATAGCAG-3’, human *53BP1* shRNA TRCN0000018865: 5’-GATACTTGGTCTTACTGGTTT-3’, human *RIF1* shRNA TRCN0000155431: 5’-CGCATTCTGCTGTTGTTGATT-3’).

### CRISPR/Cas9 gene editing

For CRISPR/Cas9-mediated gene knockout, sgRNA sequences against *Ube2d3* and *Cbx5* were designed using the Broad Institute’s GPP sgRNA Designer (https://portals.broadinstitute.org/gpp/public/analysis-tools/sgrna-design). sgRNA sequences were cloned into a pLentiCRISPRv2 plasmid according to standard protocols^52^. Lentiviral particles were packaged and transduced as described above. LentiCRISPRv2 was a gift from Feng Zhang (Addgene plasmid #52961)^52^. The following lentiviral sgRNAs were used: control non-targeting sgRNA: 5’-GACTTTGAGGAAGAGTCG-3’, mouse *Ube2d3* sgRNA: 5’-AGGACAATAATACTTGCCTT-3’, mouse *Cbx5* sgRNA: 5’-TGGACAGGCGCATGGTTAAG-3’.

### Quantitative real-time PCR

RNA was isolated using Trizol reagent (Ambion) and reverse transcribed into cDNA using AMV first-strand cDNA synthesis kit for RT–PCR (Roche) or Maxima First Strand cDNA Synthesis Kit for RT-qPCR (Thermo Fisher Scientific), according to the manufacturer’s instructions. Quantitative real-time PCR (qRT–PCR) on cDNA was performed using Power SYBR Green PCR Master Mix (Applied Biosystems) on the StepOnePlus real-time PCR system (software version 2.2.2.) or the Roche LightCycler 480 II (software version 1.5.0 sp3). The following primers were used: mouse *Ube2d3* Fw: 5’-CGTGCAATCTTATTCCTTGTCC-3’, mouse *Ube2d3* Rev: 5’-AAGGCATGCACTTCACTTCC-3’, mouse *Ube2d1* Fw: 5’-CGGGACACGCGGACACTCC-3’, mouse *Ube2d1* Rev: 5’-GATAGGCGCTGTCAGGGGGC-3’, mouse *Ube2d2* Fw: 5’-GTCGCCACCGTTTCCCACCA-3’, mouse *Ube2d2* Rev: 5’-AGGGGCTGTCATTTGGCCCC-3’, mouse *Rnf168* Fw: 5’-AGGAGCCTGAGCACCAGTATCAACA-3’, mouse *Rnf168* Rev: 5’-CAAGGCGGTCCCAGGCTCCTA-3’, mouse *Ubc13 (Ube2n)* Fw: 5’-GCCCAAGCCATAGAAACAGCGAGA-3’, mouse *Ubc13 (Ube2n)* Rev: 5’-TCAGCATCTCTCAGCAACCCGGA-3’, mouse *Trf2* Fw: 5’-GAGAGCCACCTGGATGACAC-3’, mouse *Trf2* Rev: 5’-TTCTGAGGCTGTCTGCTTGG-3’, mouse *Trip12* Fw: 5’-GGGGCCTAACTAGTGAATGGTAG-3’, mouse *Trip12* Rev: 5’-GTCTGGACTAGCACTGCGTT-3’, mouse *Ppp2ca* Fw: 5’-CCTCTGCGAGAAGGCTAAAGAA-3’, mouse *Ppp2ca* Rev: 5’-TCTCGTGATTCCCTCGGAGT-3’, mouse *Hprt* Fw: 5’-CTGGTGAAAGGACCTCTCG-3’, mouse *Hprt* Rev: 5’-TGAAGTACTCATTATAGTCAAGGGCA-3’, mouse *β-Actin* Fw: 5-CAGTAATGGCATTTGTC-3’, mouse *β-Actin* Rev: 5-CTCATCTGAGAAGACTTAAG-3’, human *GAPDH* Fw: 5’-GAAGGTGGAAGGTCGGAGTC-3’, human *GAPDH* Rev: 5’-GAAGATGGTGATGGGATTTC-3’, human *UBE2D3* Fw: 5’-CCGTCTGGCTTCGGCCTCAC-3’ and human *UBE2D3* Rev: 5’-ACACAGGCGCCTCTTCACCG-3’.

### Chromatin extraction

Cell pellets were harvested, resuspended in 2 volumes of lysis buffer (10 mM HEPES pH 7.4, 10 mM KCl, 0.05% NP-40; freshly supplemented with protease inhibitors, phosphatase inhibitors, 0.1 mM PMSF, 1 mM DTT and 10 mM Iodoacetamide) and incubated on ice for 20 min. Pellets were washed with lysis buffer, resuspended in 2 volumes of low salt buffer +0.1% Triton X100 (10 mM Tris-HCl pH 7.4, 0.2 mM MgCl_2_; freshly supplemented with protease inhibitors, phosphatase inhibitors, 0.1 mM PMSF, 1 mM DTT and 10 mM Iodoacetamide) and incubated on ice for 15 min. Pellets were resuspended in 2 volumes of HCl 0.2 N and incubated for 20 min on ice. Supernatants were neutralised with an equal volume of 1 M Tris-HCl pH 8. This final fraction contains the chromatin. Concentrations were determined by Bradford assay (Bio-Rad).

### Ubiquitination IPs in 293T

HEK 293T cells transduced with control shRNA or *UBE2D3* shRNA2 were transfected with pMSCV-GFP or pMSCV-GFP-RNF168 and pcDNA3.1(+)-HA-Ubiquitin^53^ using CaPO_4_ precipitation. Medium was refreshed 16 h after transfection. At 42 h after transfection, cells were treated for 6 h with 10 μM MG132 (Z-Leu-Leu-Leu-al, C2211, Sigma-Aldrich), washed and scraped in ice-cold PBS, centrifuged for 5 min at 1000 rpm at 4 °C and washed 2x in ice-cold PBS. Cell pellets were lysed in lysis buffer (20 mM Tris-HCl pH 8.0, 150 mM NaCl, 1% Triton X-100, 1 mM EDTA and 0.1% SDS; freshly supplemented with protease inhibitors (cOmplete, 4693124001, Roche), phosphatase inhibitors (PhosSTOP™, 4906837001, Roche), 0.1 mM PMSF, 1 mM DTT and 10 mM Iodoacetamide), sonicated for 15 min (Diagenode Bioruptor) and centrifuged for 15 min at maximum speed at 4 °C. Lysates were added to GFP-Trap beads (GFP-Trap®_MA gtma-20, Chromotek) and incubated overnight, rotating at 4 °C. Beads were washed 1x with lysis buffer and 2x with dilution buffer (10 mM Tris-HCl pH 7.5, 150 mM NaCl and 0.5 mM EDTA). Bound proteins were eluted with 2x SDS sample buffer (125 mM Tris pH 6.8, 20% glycerol, 4% SDS) + 0.04% Bromophenol blue and 10% β-mercaptoethanol, and by boiling for 10 min at 95 °C. Immunoblotting was done as described below.

### RNF168 ubiquitination IPs in HeLa

HeLa cells, transduced with a 10xHis-Ubiquitin-IRES-Puro lentiviral plasmid^54^ and selected on 1 μg ml^−1^ puromycin for 2 weeks, were seeded at 5% confluency on a 15 cm dish and let to attach overnight. Next, cells were transduced with lentivirus containing either a control shRNA or two different shRNAs targeting UBE2D3 (shControl - SHC002, shUBE2D3#1 TRCN0000038790, shUBE2D3#2 TRCN0000038792 - Mission shRNA Library, Sigma-Aldrich) at MOI 3. At 24 h after transduction, medium was replaced with fresh medium and 3 days after transduction, cells were lysed for His-Ubiquitin conjugate purification.

His-Ubiquitin conjugate purification was performed similar to as described before^55^. In brief, cells were harvested in ice-cold PBS. For total lysates (input), a small aliquot of cells was lysed in SNTBS (2% SDS, 1% N-P40, 50 mM Tris pH 7.5, and 150 mM NaCl). The remaining part of the sample were lysed in 3 ml of Guanidinium buffer (6 M guanidine-HCl, 0.1 M Na2HPO4/NaH2PO4, 10 mM Tris, pH 7.8). For His-Ubiquitin-conjugates purification, cell lysates were homogenised by two rounds of sonication (5 s, 80% Amplitude) using a Misonix Sonicator 3000 (Cole-Parmer). Protein concentrations were determined using Pierce BCA Protein Assay Reagent (Thermo Fisher Scientific) and samples were equalised accordingly. Subsequently, 50 mM imidazole and 5 mM β-mercaptoethanol were added. Enrichment of His10-Ub conjugates was performed using nickel-nitrilotriacetic acid-agarose beads (Ni-NTA) (Qiagen). 60 μl of Ni-NTA beads, equilibrated beforehand with Guanidinium buffer, were added to each sample and incubated overnight at 4 °C under rotation. After incubation, beads were washed using wash buffers A–D. Wash buffer A (6 M guanidine-HCl pH 7.8, 10 mM imidazole pH 8.0, 5 mM β-mercaptoethanol, and 0.2% Triton X-100). Wash buffer B (8 M urea, 0.1 M Na_2_HPO_4_/NaH_2_PO_4_, pH 8.0, 0.01 M Tris-HCl pH 8.0, 10 mM imidazole pH 8.0, 5 mM β-mercaptoethanol, and 0.1% Triton X-100), Wash buffer C (8 M urea, 0.1 M Na_2_HPO_4_/NaH_2_PO_4_, pH 6.3, 0.01 M Tris-HCl pH 6.3, 10 mM imidazole pH 7.0, 5 mM β-mercaptoethanol). Wash buffer D: (8 M urea, 0.1 M Na_2_HPO_4_/NaH_2_PO_4_, pH 6.3, 0.01 M Tris-HCl, pH 6.3, no imidazole, 5 mM β-mercaptoethanol). The samples were eluted twice using 60 μl elution buffer in 7 M urea, 0.1 M NaH_2_PO_4_/Na_2_HPO_4_, 0.01 M Tris/HCl, pH 7.0, and 500 mM imidazole pH 7.0.

For immunoblotting, samples were run on pre-casted NuPAGE 4–12% Bis-Tris gradient gels (Invitrogen, Thermo Fisher Scientific) according to vendor instructions, using MOPS buffer. Transfer was performed by electroblotting in transfer buffer (50 mM Tris, 0.4 M Glycine, 10% Methanol) at 350 mA for 200 min to Amersham™ Protran™ Premium 0.45 μm Nitrocellulose membrane.

### In vivo PP2A ubiquitination assay

HEK 293T cells were grown on 10 cm plates until ~70% confluency, and transfected with the indicated plasmids using CaPO_4_ precipitation. 48 h post transfection, cells were washed twice with PBS and lysed with 150 μl of lysis buffer containing 50 mM HEPES pH 7.3, 250 mM NaCl, 0.5% Igepal and 1% SDS, further supplemented with 10 mM N-ethylmaleimide, EDTA free protease inhibitors (cOmplete, 4693159001, Roche) and phosphatase inhibitors (PhosSTOP^TM^, 4906837001, Roche). The lysates were sonicated twice with a tip sonicator at 80% amplitude for 20 s with 5 s ON and 5 s OFF cycles. Thereafter, the lysates were diluted by adding 1350 μl of lysis buffer without SDS, to reduce the final SDS concentration to 0.1%, mixed briefly and centrifuged at 15,294 *g* for 15 min. The supernatants were transferred to new tubes and protein concentration was determined by standard Pierce BCA protein assay (Thermo Fisher Scientific). Equal amounts of protein for each sample were used for the immunoprecipitation. Each of the sample lysates was incubated with 2 μl of anti-PP2A, C subunit, clone 1D6 antibody (05-421, Sigma-Aldrich/Millipore) and kept for rotation in the cold room overnight. The next day, Dynabeads^TM^ Protein G (10004D, Invitrogen, Thermo Fisher Scientific) were added and kept for further 1 h in the cold room with gentle rotation. Thereafter, beads were extensively washed with 0.1% SDS containing lysis buffer and eventually boiled with 2x LDS (NuPAGE) sample buffer and loaded onto SDS PAGE gels.

### Immunoblotting

Whole-cell lysates were prepared by scraping cells in SDS sample buffer (125 mM Tris pH 6.8, 20% glycerol, 4% SDS), shearing the lysates with a 25 G needle and boiling for 10 min at 95 °C. Alternatively, cells scraped in SDS sample buffer were sonicated with a tip sonicator at 20% amplitude for 10 s with 1 s intervals. Sonicated samples were centrifuged at 15,294 *g* for 10 min at 4 °C and supernatants were collected. Protein concentration was determined by standard Pierce BCA protein assay (Thermo Fisher Scientific). Equal amounts of protein were separated on precast NuPAGE 4–12% Bis-Tris or 3–8% Tris-acetate gels (Invitrogen, Thermo Fisher Scientific). Immunoblotting was done according to standard methods. IRDye800CW- and IRDye680-labelled secondary antibodies were used for detection on the Odyssey and Odyssey CLx Infra-red imagers (LI-COR), using ImageStudio 5.2.5 software. Horseradish peroxidase (HRP)-conjugated secondary antibodies were used for detection by enhanced chemiluminescence (Supersignal and Supersignal West Pico Plus, Thermo Fisher Scientific), either on film (Amersham Hyperfilm MP), on a Chemidoc XRS + 4.0.1 (Bio-Rad) or a G: box mini Syngene (with Genesys v1.6.9.0 software).

Primary antibodies used were against UBE2D3 (Y-25, sc-100618, SCBT, 1:500; 11677-1-AP, Proteintech, 1:500; 4330S, CST, 1:500 and A615, Boston Biochem, 1:2000), KAP1 (22553, Abcam, 1:1000), phospho-Kap1 S824 (A300 767A, Bethyl, 1:1000), 53BP1 (NB100-305, Novus, 1:500 and A300-272A, Bethyl, 1:2000), phospho-ATMS1981 (4526, CST, 1:1000), phospho-H2AX S139 (5636, Millipore, 1:1000), CHK2 (611570, BD, 1:500), c-myc (9E10, sc-40, SCBT, 1:250), HA (MMS-101R, Covance, 1:1000), TRF2 (NB110-57130, Novus, 1:500), RNF8 (sc-133971, SCBT, 1:250), MAD2L2 (14, sc-135977, SCBT, 1:500), GFP IgG fraction (A11122, Thermo Fisher Scientific, 1:1000), FLAG M2 (F1804, Sigma-Aldrich, 1:1000), Histone H3 (ab1791, Abcam, 1:10,000), hRNF168 (ABE367, Millipore, 1:500), hRNF168 (ABE467, Merck-Millipore, 1:1000), mRnf168 (gift from D. Durocher, 1:1000), hRIF1 (A300-569A, Bethyl, 1:1000), mRIF1 (gift from S. Boulton and R. Chapman, 1:1000), Ligase 4 (H-300, sc-28232, SCBT, 1:300; NB110-57379, Novus, 1:500), FK2 (04-263, Millipore, 1:2000), HP1α (2616S, CST, 1:1000), Ubiquitin (P4D1, sc-8017, SCBT, 1:1000), HDAC1 (PA1-860, Thermo Fisher Scientific, 1:1000), PP2A C subunit, clone 1D6 antibody (05-421, Sigma-Aldrich/Millipore, 1:500), CDK4 (C-22, sc-260, SCBT, 1:500), HSP90 α/β (H-114, sc-7947, SCBT, 1:1000), γ-tubulin (T6557, Sigma-Aldrich, 1:10,000) β-actin (A5316, Sigma-Aldrich, 1:10,000), β-catenin (610154, BD, 1:10,000) and GAPDH (PA1-987, Thermo Fisher Scientific, 1:1000). HSP90 α/β, γ-tubulin, β-actin, β-catenin and GAPDH were used for loading controls.

### Immunofluorescence

For immunofluorescence, cells were grown on 8-well chamber slides (Lab-Tek) and in case of FK2 (conjugated ubiquitin) detection first pre-extracted for 3–5 min with 0.5% Triton/PBS on ice, then fixed for 10 min in 4% paraformaldehyde in PBS. Cells were permeabilised for 5–10 min in 0.5% Triton/PBS, washed twice in PBS, incubated for 1 h in blocking solution (0.02%Triton, 5% NGS, 5% FCS in PBS) and overnight at 4 °C with primary antibodies in blocking solution. Primary antibodies used were against phospho-ATMS1981 (4526, CST 1:1000), phospho-H2AX Ser 139 (05-636, Millipore and 2577S, CST, 1:500), 53BP1 (A300-272A, Bethyl, 1:2000), mRIF1 (gift from S. Boulton and R. Chapman,1:500), phospho-DNA-PKcs Ser 2056 (ab18192, Abcam, 1:500), GFP IgG fraction (A11122, Thermo Fisher Scientific, 1:500) and FK2 (04-263, Millipore, 1:5000). Cells were washed three times with 0.02% Triton/PBS, followed by incubation with Alexa Fluor 488 or 568 goat anti-mouse or anti-rabbit IgG secondary antibodies (Invitrogen) in blocking solution for 1 h. After four washes in 0.02% Triton/PBS, slides were mounted in Vectashield (Vector Laboratories) containing DAPI. Confocal fluorescence images were obtained on a Leica SP5 confocal system equipped with an Ar, Kr and HeNe laser system. Images were taken with a x63 NA 1.32 oil objective and standard LAS-AF software. Possible crosstalk between fluorochromes was avoided by careful selection of imaging conditions. A minimum of 100 cells per condition per experiment was analysed and foci were counted on maximum-intensity projection using an automatic and objective analysis as described before^56^. Foci for phospho-H2AX, phospho-ATM, 53BP1, RIF1 and FK2 in MEFs were captured using the Metafer4/MetaCyte v3.11.113 platform (MetaSystems) equipped with an AxioImager Z2 microscope (Carl Zeiss). Images of random selections of cells were acquired with an EC ‘Plan-Neofluar’ x40/0.75 objective. Analysis was done using MetaCyte software, carefully fine-tuned for each antibody. A minimum of 500 cells was analysed per condition per experiment.

### Metaphase chromosome analysis

Cell collection, preparation of metaphase spreads and telomere FISH with a FITC-OO-(CCCTAA)_3_ peptide nucleic acid custom probe (Biosynthesis) or Alexa488-labelled C-rich Telomere Probe (Eurogentec) for metaphase chromosome analysis was done as described^4^. For metaphase chromosome preparation from doxycycline-inducible shTRF2 HeLa cells (Fig. 2c), TRF2 depletion was induced with 1 μg/ml doxycycline (+DOX) and metaphases were harvested upon 96 h treatment. Digital images of metaphases were captured using the Metafer4/MSearch automated metaphase finder system (MetaSystems) equipped with an AxioImager Z2 microscope (Carl Zeiss). After scanning metaphase preparations at x10 magnification, high-resolution images of metaphases were acquired using a ‘Plan-Apochromat’ x63/1.40 oil objective. Chromosome fusions were quantified from >30 metaphases (or >1500 chromosomes) per experimental condition.

### Pulse-field gel electrophoresis and in-gel detection of telomeric DNA

Analysis of 3’ single-stranded G-overhangs at mouse telomeres was performed as described^4^ by pulsed-field gel electrophoresis and in-gel hybridisation of a ^32^P-labelled telomeric repeat (CCCTAA)_4_ oligonucleotide to native DNA. In brief, TRF2ts MEFs were collected at the permissive temperature as well as after growth at the non-permissive temperature. Agarose plugs were prepared containing 1 × 10^6^ cells per plug and digested overnight with 1 mg ml^−1^ proteinase K at 50 °C. Before loading on a 1% agarose gel, plugs were digested overnight with 60U of MboI enzyme per plug. After running the gel on a CHEF-DR III pulsed-field gel electrophoresis system (Bio-Rad), the gel was dried, pre-hybridised in Church Mix containing 7% SDS and hybridised overnight with a ^32^P-labelled (CCCTAA)_4_ probe, all under non-denaturing conditions. Signal was captured on a phosphor screen, detected with a Fujifilm FLA-3000 R laser imaging scanner and analysed with AIDA Image Analyzer software version 3.40. To confirm that the G-overhang signal detected with this method was indeed derived from 3’ single-stranded telomeric TTAGGG repeats, digestion with 3’ exonuclease and hybridisation with a ^32^P-labelled (TTAGGG)_4_ probe specific for the C-rich strand was performed on separate plugs. After capturing the hybridisation signal from single-stranded telomeric DNA, the gel was denatured and re-hybridised with the same probes to obtain a total telomere signal for the purpose of quantification.

### Cycloheximide assay

To assess RNF168 protein stability, HEK 293T cells transduced with either control or *UBE2D3* shRNA were seeded on 6 cm dishes and treated with 50 μg/ml cycloheximide. Cells were harvested at the indicated timepoints, lysates were separated on precast 4–12% Bis-Tris gels (Invitrogen), followed by immunoblotting.

### Random plasmid integration assay

For random plasmid integration assays 500,000 U2OS cells (ATCC) were seeded in 6 cm dishes 24 h before transfection with 3.9 μg of NdeI-linearised pMSCVblas-GFP and 0.1 μg of mCherry plasmids using ViaFect (Promega). Transfected cells were trypsinised the following day and seeded in 10 cm dishes at different densities for colony formation. Selection was initiated the following day with blasticidin at 5 μg ml^−1^. Cells were fixed with 4% paraformaldehyde 10–14 days after plating and stained with 0.1% crystal violet. Colony counting was performed on a Col-Count machine (Oxford Optronix) with Col-Count v3.3 software and values were normalised for plating and transfection efficiencies.

### PP2A activity assay

PP2A activity was measured using a PP2A Immunoprecipitation Phosphatase Assay Kit (17-313, Sigma-Aldrich/Millipore). Briefly, control and *Ube2d3* shRNA transduced TRF2ts MEFs were incubated at 32 °C or 37 °C for 3 h, and RIDDLE syndrome patient cells were left untreated or harvested 30 min after exposure to 3 Gy. Cells were washed 2x with ice-cold TBS, scraped in ice-cold TBS supplemented with Halt Protease Inhibitor Cocktail (78430, Thermo Fisher Scientific) and collected. Subsequently, cells were sonicated on ice with a tip sonicator at 40% amplitude for 20 s with 1 s intervals. Lysates were centrifuged at 15,294 *g* for 10 min. Supernatants were collected and protein concentration was determined by standard Pierce BCA protein assay (Thermo Fisher Scientific). 500 μg sample was diluted with 500 μl TBS and used to perform PP2A immunoprecipitation, according to the instructions of the manufacturer of the kit. Briefly, anti-PP2A, C subunit, clone 1D6 antibody (05-421, Sigma-Aldrich/Millipore) and Protein A agarose beads (16-125, Sigma-Aldrich/Millipore) were mixed, divided over all samples and rotated for 2 h at 4 °C. The beads were washed 3x with TBS and 2x with pNPP Ser/Thr Assay Buffer (20-179, Sigma-Aldrich/Millipore). The obtained PP2A-IP samples were used in colorimetric phosphatase assays according to the manufacturer’s instructions. Absorbance values of samples were measured in triplicate and corrected to the negative control (mouse IgG). SDS sample buffer (125 mM Tris pH 6.8, 20% glycerol, 4% SDS) + 0.04% Bromophenol blue and 10% β-mercaptoethanol was added to the beads and bound proteins were eluted by boiling for 5 min at 95 °C, followed by protein gel electrophoreses and immunoblotting.

### Proteome and phosphoproteome analysis

#### Sample preparation

Samples were prepared from TRF2ts MEFs transduced with shScramble (control) or *Ube2d3* sh1, or treated with LB100, each with/without telomere uncapping at 37 °C for 3 h. Samples were processed and analysed in biological triplicates *(n* = *3)*.

For protein digestion, frozen cell pellets were lysed in boiling Guanidine (GuHCl) lysisbuffer as described by ref. ^57^. Protein concentration was determined with a Pierce Coomassie (Bradford) Protein Assay Kit (Thermo Scientific), according to the manufacturer’s instructions. After dilution to 2 M GuHCl, aliquots corresponding to at least 0.4 mg of protein were digested twice (4 h and overnight) with trypsin (Sigma-Aldrich) at 37 °C, enzyme/substrate ratio 1:75. Digestion was quenched by the addition of FA (final concentration 5%), after which the peptides were desalted on a Sep-Pak C18 cartridge (Waters, Massachusetts, USA). From the eluates, aliquots were collected for proteome analysis, the remainder being reserved for phosphoproteome analysis. Samples were vacuum dried and stored at −80 °C until LC-MS/MS analysis or phosphopeptide enrichment.

#### Phosphopeptide enrichment

Phosphorylated peptides were enriched from 0.4 mg total peptides using r2p2 as described by ref. ^58^, with the exceptions that it was performed by hand and the dried eluates were reconstituted in 15 μl of 2% formic acid.

#### Mass spectrometry: RP-nanoLC-MS/MS

Prior to mass spectrometry analysis, the peptides were reconstituted in 2% formic acid. Peptide mixtures were analysed by nanoLC-MS/MS on an Orbitrap Exploris 480 Mass Spectrometer equipped with an EASY-NLC 1200 system (Thermo Scientific). Samples were directly loaded onto the analytical column (ReproSil-Pur 120 C18-AQ, 2.4 μm, 75 μm × 500 mm, packed in-house). Solvent A was 0.1% formic acid/water and solvent B was 0.1% formic acid/80% acetonitrile.

For single-run proteome, samples were eluted from the analytical column at a constant flow of 250 nl/min in a 90-min gradient, containing a 78-min linear increase from 6% to 30% solvent B, followed by a 12-min wash at 90% solvent B. For the phosphoproteome, a 85-min gradient was employed containing a linear increase from 5% to 32% solvent B, followed by a 15-min wash.

Peptide mixtures were analysed by nanoLC-MS/MS on an Orbitrap Exploris 480 Mass Spectrometer equipped with an Evosep One LC system. Peptides were separated using the pre-programmed gradient (Extended method, 88 min gradient) on an EV1137 (Evosep) column with an EV1086 (Evosep) emitter.

#### Data analysis

For proteome data, raw data were analysed by DIA-NN (version 1.8)^59^ without a spectral library and with ‘Deep learning’ option enabled. The Swissprot *Mus musculus* database (17,125 entries, release 2022_08) was added for the library-free search. The Quantification strategy was set to Robust LC (high accuracy) and MBR option was enabled. The other settings were kept at the default values. The protein groups report from DIA-NN was used for downstream analysis in Perseus (version: 2.0.10.0)^60^. Values were Log2-transformed, after which proteins were filtered for at least 75% valid values in at least one sample group. Missing values were replaced by imputation based a normal distribution using a width of 0.3 and a minimal downshift of 2.4. Differentially expressed proteins were determined using a Student’s t-test (minimal threshold: –log(*p*-value) ≥ 1.3 and [x-y] ≥ 0.5 | [x-y] ≤ −0.5).

For phosphoproteome, data (RAW files) were analysed by MaxQuant (version 2.4.2.0)^61^ using standard settings. MS/MS data were searched against the *Mus musculus* Swissprot database (17,125 entries, release 2022_08) complemented with a list of common contaminants and concatenated with the reversed version of all sequences. The maximum allowed mass tolerance was 4.5 ppm in the main search and 20 ppm for fragment ion masses. False discovery rates for peptide and protein identification were set to 1%. Trypsin/P was chosen as cleavage specificity allowing two missed cleavages. Carbamidomethylation (C) was set as a fixed modification, while oxidation (M) and phosphorylation (S, T, Y) were used as variable modifications. The LFQ intensities were Log2-transformed in Perseus (version 2.0.10.0), after which the phosphosites were filtered for at least 75% valid values in at least one condition. Missing values were replaced by imputation based on a normal distribution using a width of 0.3 and a downshift of 1.8. Differentially regulated phosphosites were determined using a t-test (minimal threshold: –log(*p*-value) ≥ 1.3 and [x-y] ≥ 1 | [x-y] ≤ −1).

### Reporting summary

Further information on research design is available in the Nature Portfolio Reporting Summary linked to this article.

### Supplementary information

Supplementary Information

Peer Review File

Reporting Summary

### Source data

Source Data

## Data Availability

Materials are available from the corresponding authors upon reasonable request. Source data are provided with this paper. The mass spectrometry proteomics data generated in this study have been deposited in the ProteomeXchange Consortium via the PRIDE^62^ partner repository under accession code PXD044410. All data generated this study are included in this published article and its supplementary information files and are available from the corresponding author upon request. Source data are provided with this paper.
