# Supplementary material for: UBE2D3 facilitates NHEJ by orchestrating ATM signalling through multi-level control of RNF168
Source: Nat Commun. 2024 Jun 12;15:5032. doi: 10.1038/s41467-024-49431-6 (PMC11169547; doi:10.1038/s41467-024-49431-6)
Supplement: Supplementary file 1 — Supplementary Information [file 41467_2024_49431_MOESM1_ESM.pdf]

Supplementary Information

UBE2D3 facilitates NHEJ by orchestrating ATM signalling through multi-level control of RNF168

Zeliha Yalcin[1#], Shiu Yeung Lam[1#], Marieke H. Peuscher[1], Jaco van der Torre[1], Sha Zhu[1], Prasanna V. Iyengar[1], Daniel Salas-Lloret[2], Inge de Krijger[1], Nathalie Moatti[1], Ruben van der Lugt[1], Mattia Falcone[1], Aurora Cerutti[1], Onno B. Bleijerveld[3], Liesbeth Hoekman[3], Román González-Prieto[2,4,5], Jacqueline J. L. Jacobs[1*]

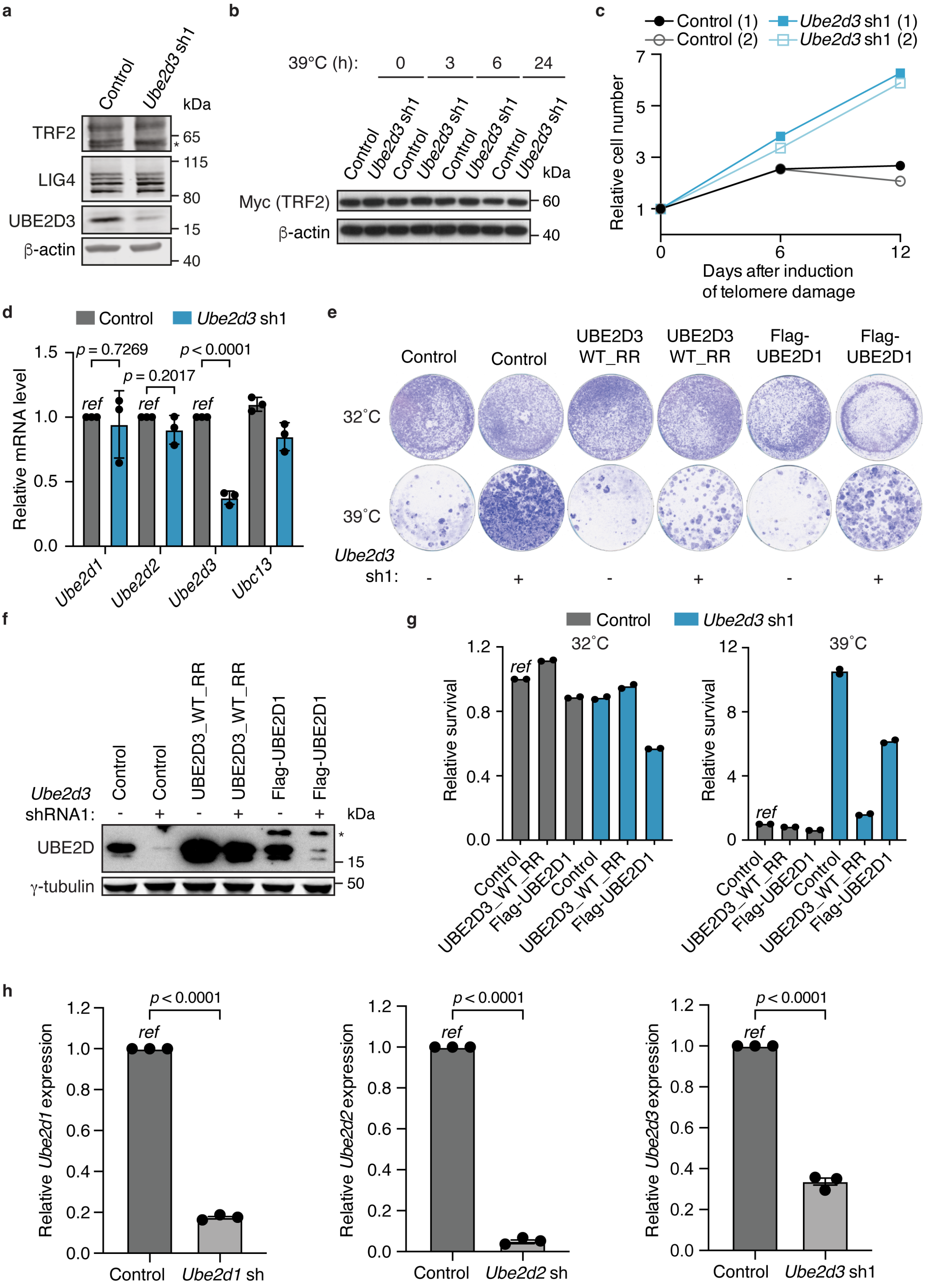
a
Control
Ube2d3 sh1
kDa
TRF2
65
*
LIG4
115
80
UBE2D3
15
β-actin
40
b
39°C (h):
0
3
6
24
Control
Ube2d3 sh1
kDa
Myc (TRF2)
60
β-actin
40
c
Control (1)
Control (2)
Ube2d3 sh1 (1)
Ube2d3 sh1 (2)
Relative cell number
Days after induction of telomere damage
d
Control
Ube2d3 sh1
Relative mRNA level
p = 0.7269
p = 0.2017
p < 0.0001
ref
Ube2d1
Ube2d2
Ube2d3
Ubc13
e
Control
UBE2D3 WT_RR
Flag-UBE2D1
32˚C
39˚C
Ube2d3 sh1:
-
+
f
Ube2d3 shRNA1:
Control
UBE2D3_WT_RR
Flag-UBE2D1
kDa
UBE2D
*
15
γ-tubulin
50
g
Control
Ube2d3 sh1
32˚C
39˚C
Relative survival
ref
Control
UBE2D3_WT_RR
Flag-UBE2D1
h
Relative Ube2d1 expression
Relative Ube2d2 expression
Relative Ube2d3 expression
p < 0.0001
ref
Control
Ube2d1 sh
Ube2d2 sh
Ube2d3 sh1

Supplementary Figure 1. UBE2D3 does not affect TRF2 stability and promotes telomere uncapping induced crisis. **a**, Immunoblotting for TRF2, UBE2D3 and LIG4 in TRF2ts MEFs transduced with control or *Ube2d3* shRNA1, used in Fig. 1b. **b**, Immunoblotting for exogenous myc-TRF2ts levels in control and UBE2D3-depleted TRF2ts MEFs upon telomere uncapping at 39°C. Representative blots from *n=2* independent experiments are shown. **c**, Growth curve of control and *Ube2d3* knockdown TRF2ts cells clone B17 upon induction of telomere uncapping from *n=2* independent experiments (labelled (1) and (2)). **d**, q-RT PCR analysis of *Ube2d1*, *Ube2d2*, *Ube2d3* and *Ubc13* mRNA levels in *Ube2d3* knockdown TRF2ts MEFs (*n=3* independent experiments for *Ube2d1*, *Ube2d2* and *Ube2d3*, *n=3* technical replicates for *Ubc13*; mean ± SD). Statistical significance was calculated using the two-tailed Student's *t*-test. **e,** Survival assay of TRF2ts MEFs with knockdown of *Ube2d3*, complemented with UBE2D3_WT RR or Flag-UBE2D1. Representative plates from *n=2* independent experiments are shown. **f**, Immunoblotting for UBE2D3 RR and Flag-UBE2D1 expression in TRF2ts MEFs used in e; the asterisk indicates Flag-UBE2D1. **g**, Quantification of the survival assay shown in e (*n=2* technical replicates). **h**, q-RT PCR analysis of *Ube2d1*, *Ube2d2* and *Ube2d3* mRNA levels in TRF2ts MEFs used in Fig. 1d (*n=3* independent experiments; mean ± SEM; two-tailed Student's *t*-test). *Ref* = reference. Source data are provided as a Source Data file.

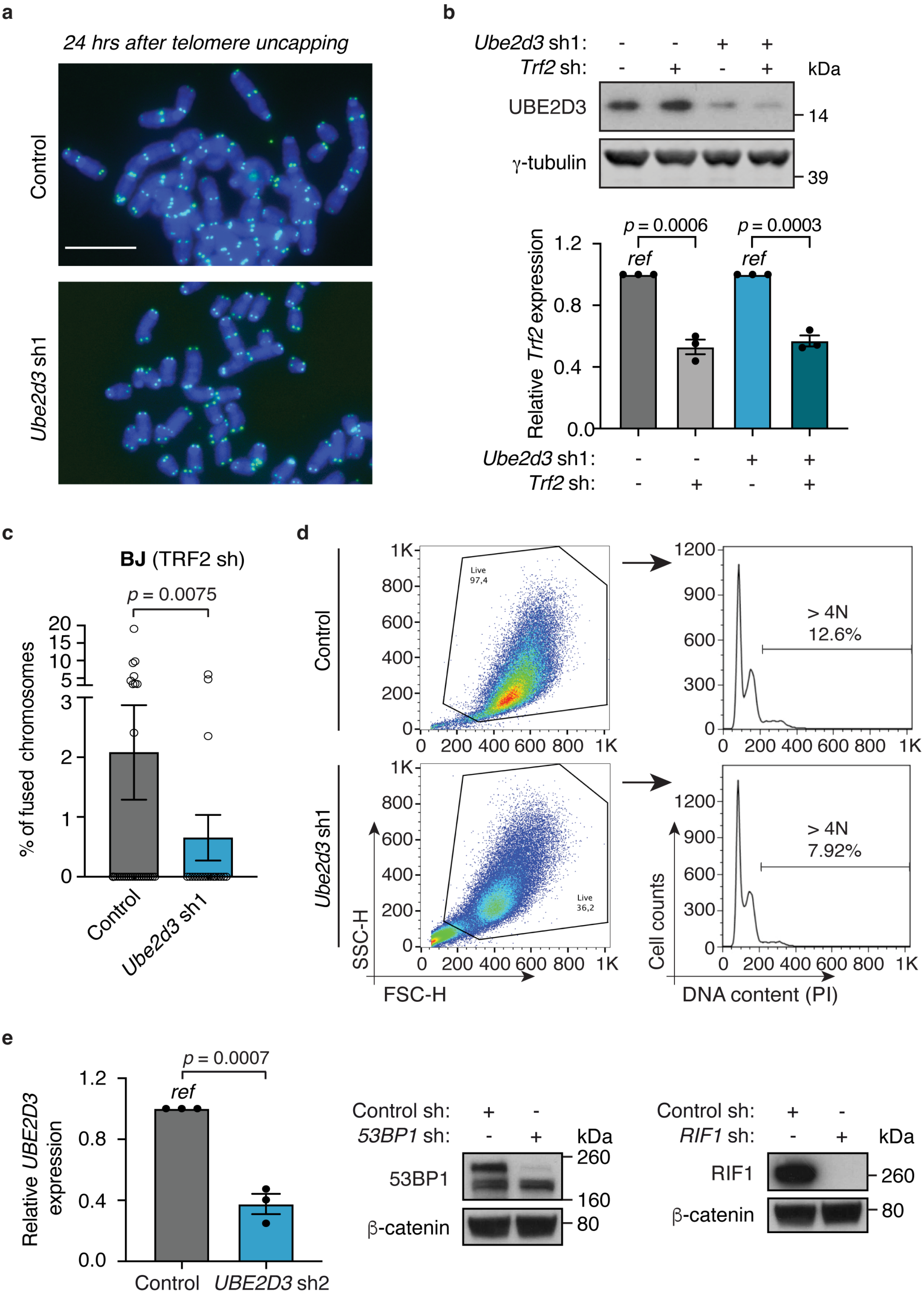
a
24 hrs after telomere uncapping
Control
Ube2d3 sh1
b
Ube2d3 sh1: - - + +
Trf2 sh: - + - +
kDa
UBE2D3
14
γ-tubulin
39
p = 0.0006
p = 0.0003
ref
ref
Relative Trf2 expression
1.2
0.8
0.4
0.0
Ube2d3 sh1: - - + +
Trf2 sh: - + - +
c
BJ (TRF2 sh)
p = 0.0075
% of fused chromosomes
20
15
10
5
3
2
1
0
Control
Ube2d3 sh1
d
Control
Ube2d3 sh1
Live
97,4
Live
36,2
1K
800
600
400
200
0
0 200 400 600 800 1K
SSC-H
FSC-H
1200
900
600
300
0
0 200 400 600 800 1K
> 4N
12.6%
> 4N
7.92%
Cell counts
DNA content (PI)
e
p = 0.0007
ref
Relative UBE2D3 expression
1.2
0.8
0.4
0.0
Control
UBE2D3 sh2
Control sh: + -
53BP1 sh: - +
kDa
53BP1
260
160
β-catenin
80
Control sh: + -
RIF1 sh: - +
kDa
RIF1
260
β-catenin
80

Supplementary Figure 2. UBE2D3 promotes telomere fusion and aneuploidy upon telomere uncapping by TRF2 inactivation. a, Representative metaphase spreads of TRF2ts MEFs transduced as indicated, collected after 24 h at 39°C and stained for DAPI (blue) and telomere FISH (green) (*n=4* independent experiments). Scale bar represents 10 μm. **b**, Immunoblotting for UBE2D3 and q-RT PCR analysis for *Trf2* mRNA levels in the WT MEFs from Fig. 2b. Representative blots and the mean ± SEM from *n=3* independent experiments are shown. Statistical analysis by two-tailed Student's *t*-test. **c**, Quantification of chromosome fusions in control and UBE2D3-depleted SV40-immortalised human BJ fibroblasts, at 6 days after expression of *Trf2* shRNA (*n=29* metaphases for control and *n=20* metaphases for *Ube2d3* sh1; mean ± SEM; chi-squared test). **D**, Example of gating strategy for aneuploidy (>4N DNA content) analysis by PI staining and flow cytometry in control and UBE2D3-depleted TRF2ts MEFs after 48 hours of telomere uncapping, used in Fig. 2d. **e**, Verification of shRNA-mediated knock-down in U2OS cells used in Fig. 2g. Left: q-RT PCR analysis of *UBE2D3* mRNA levels (*n=3* independent experiments; mean ± SEM; two-tailed Student's *t*-test). Right: immunoblotting for 53BP1 and RIF1. Representative blots from *n=3* independent experiments for 53BP1 and *n=2* independent experiments for RIF1 are shown. *Ref* = reference. Source data are provided as a Source Data file.

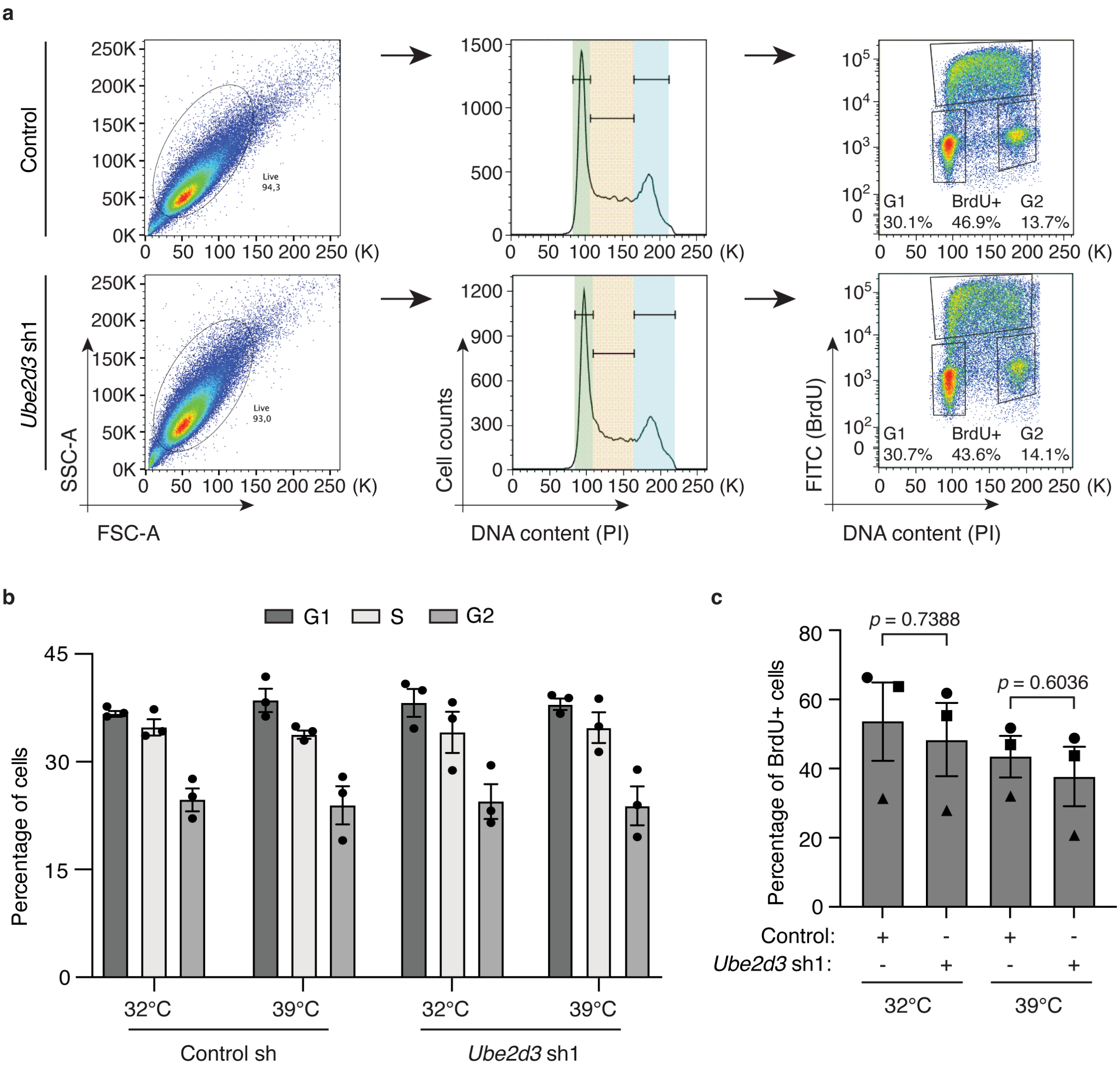

Supplementary Figure 3. UBE2D3 depletion impairs NHEJ without altering cell cycle distribution. a, Example of gating strategy for cell cycle analysis of TRF2ts control and UBE2D3-depleted cells upon 12 hours of telomere uncapping at 39°C, used in Supplementary Figure 3b,c. **b,c**, TRF2ts control cells or transduced with *Ube2d3* shRNA1 were grown at 32°C or uncapped for 12 h at 39°C. Cell cycle phase analysis by FACS was based on (**b**) propidium iodide (PI) staining and (**c**) 1h incubation with BrdU, followed by detection of BrdU incorporation and propidium iodide staining for DNA content. The mean ± SEM from *n=3* independent experiments is shown. Statistical analysis by two-tailed Student's *t*-test (Control vs *Ubed2d3* sh1). No statistically significant changes were detected (**b**), all *p*-values > 0.05. The different symbols (dot, square and triangle) represent *n=3* biologically independent experiments (**c**). Source data are provided as a Source Data file.

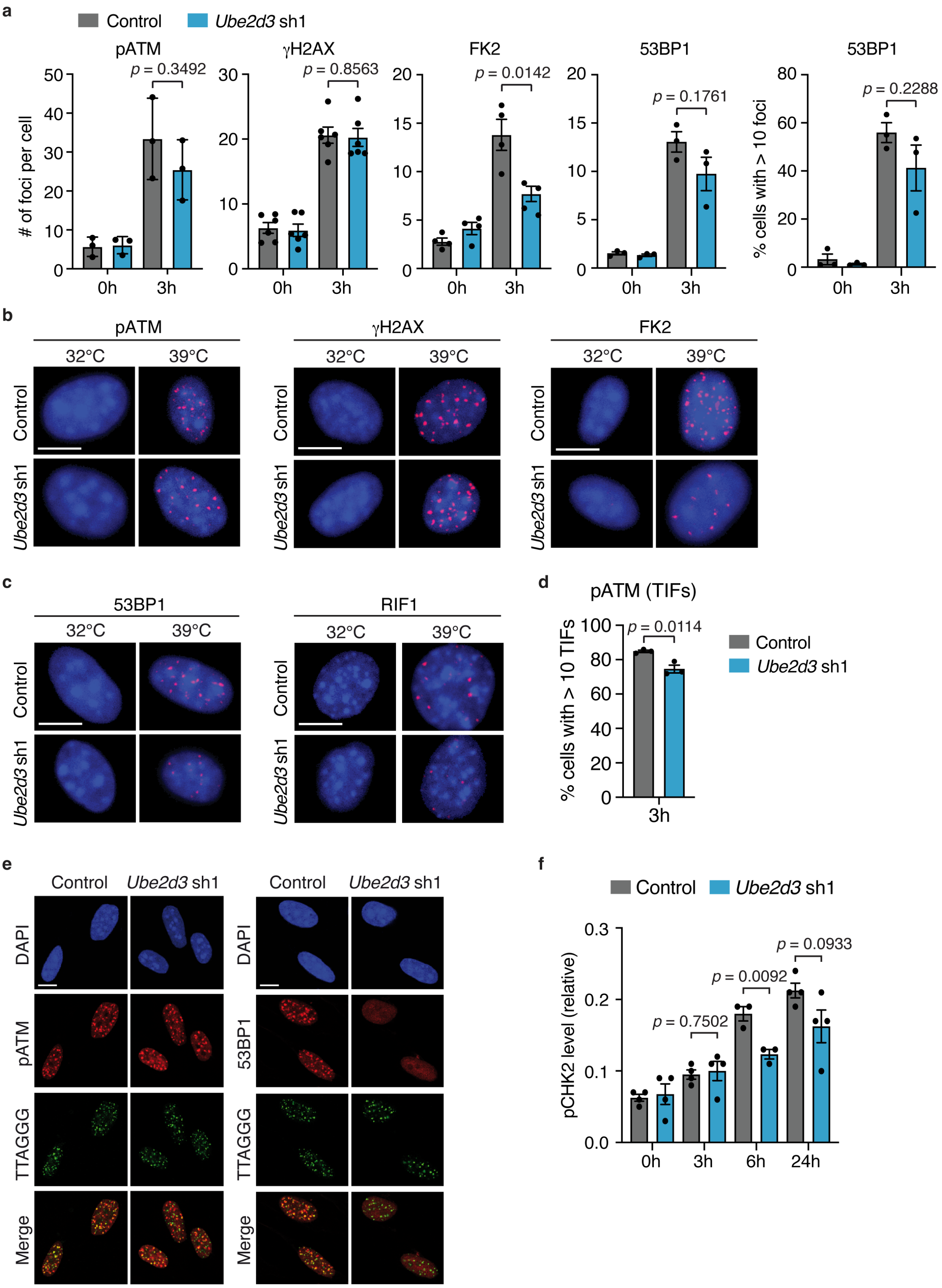

a
Control
Ube2d3 sh1
pATM
γH2AX
FK2
53BP1
53BP1
# of foci per cell
% cells with > 10 foci
p = 0.3492
p = 0.8563
p = 0.0142
p = 0.1761
p = 0.2288
0h
3h
b
pATM
γH2AX
FK2
32°C
39°C
Control
Ube2d3 sh1
c
53BP1
RIF1
32°C
39°C
Control
Ube2d3 sh1
d
pATM (TIFs)
% cells with > 10 TIFs
p = 0.0114
Control
Ube2d3 sh1
3h
e
Control
Ube2d3 sh1
DAPI
pATM
53BP1
TTAGGG
Merge
f
Control
Ube2d3 sh1
pCHK2 level (relative)
p = 0.7502
p = 0.0092
p = 0.0933
0h
3h
6h
24h

Supplementary Figure 4. UBE2D3 is dispensable for telomere damage recognition, but promotes CHK2 phosphorylation and ubiquitin conjugation and 53BP1 accumulation at DDR foci and telomeres. a, Quantification of pATM, γH2AX, FK2 and 53BP1 foci in TRF2ts MEFs transduced as indicated and uncapped for 0 or 3 h at 39°C (pATM and 53BP1: mean ± SEM from *n=3* independent experiments; γH2AX: mean ± SEM from *n=6* independent experiments; FK2: mean ± SEM from *n=4* independent experiments). Statistical analysis by two-tailed Student's *t*-test. **b,c**, Representative images of endogenous pATM (*n=3* independent experiments), γH2AX (*n=6* independent experiments), FK2 (*n=4* independent experiments), 53BP1 (*n=3* independent experiments) and RIF1 (*n=3* independent experiments) foci in TRF2ts MEFs transduced as indicated at 32°C or upon 3 h of telomere uncapping at 39°C. DNA was stained with 4',6-diamidino-2-phenylindole (DAPI). Original magnifications, X40. Scale bars represent 10 μm. **d**, Quantification of pATM foci at telomeres (TIFs) in TRF2ts MEFs upon telomere uncapping for 3 h at 39°C (*n=3* independent experiments; mean ± SEM; two-tailed Student's *t*-test). **e**, Representative images of immunofluorescence FISH (IF-FISH) detection of pATM (*n=3* independent experiments), 53BP1 (*n=5* independent experiments) and telomere repeats (TTAGGG) in TRF2ts MEFs uncapped for 3 h at 39°C. Scale bars represent 10 μm. **f**, Quantification of pCHK2 protein levels in whole cell extracts (WCE) upon telomere uncapping. The mean ± SEM from *n=4* independent experiments is shown (except for the 6h timepoint, which is *n=3*). Statistical significance was calculated using the two-tailed Student's *t*-test. Source data are provided as a Source Data file.

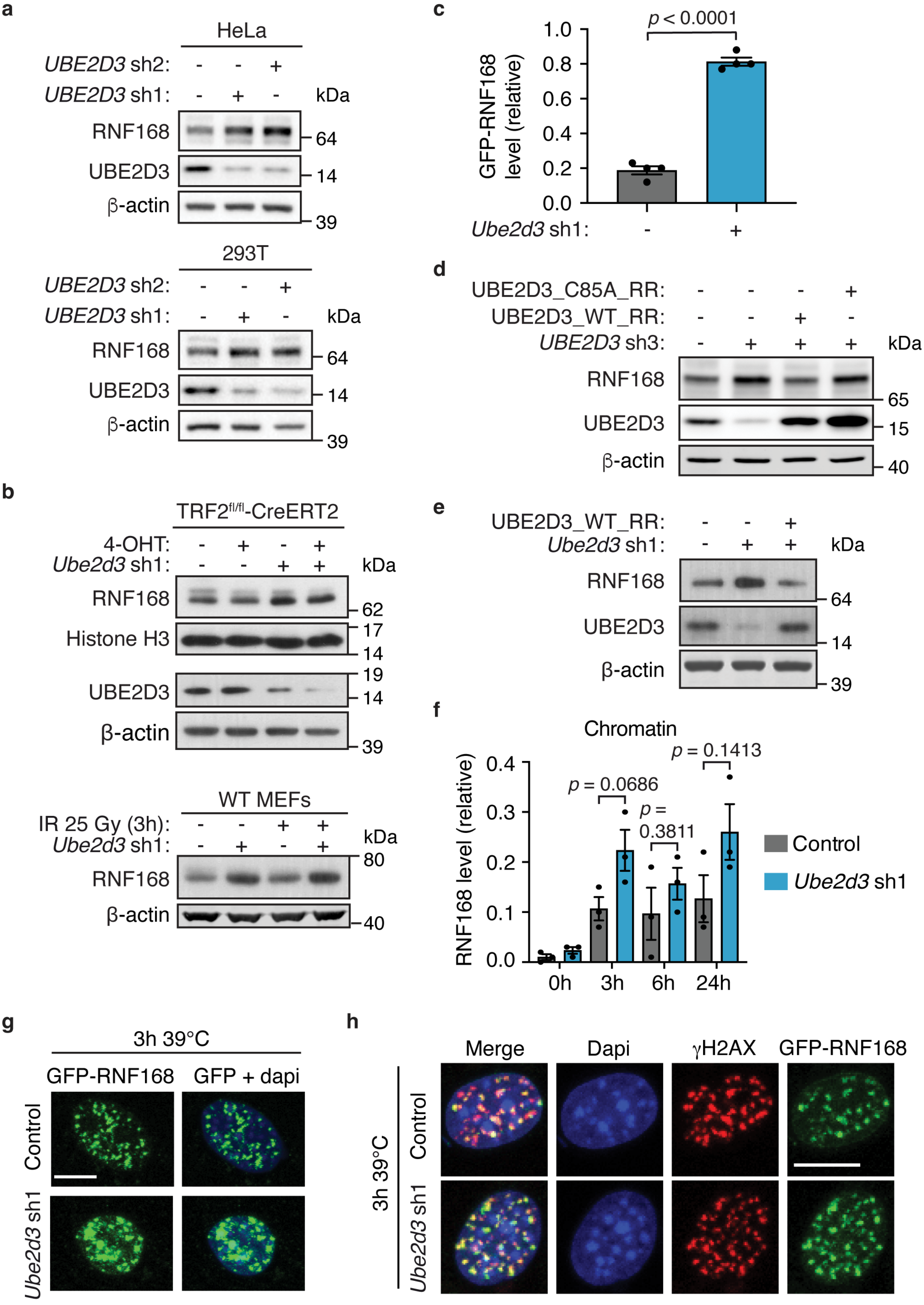
a
HeLa
UBE2D3 sh2:
UBE2D3 sh1:
kDa
RNF168
UBE2D3
β-actin
64
14
39
293T
b
TRF2fl/fl-CreERT2
4-OHT:
Ube2d3 sh1:
Histone H3
62
17
19
WT MEFs
IR 25 Gy (3h):
80
40
c
p < 0.0001
GFP-RNF168 level (relative)
Ube2d3 sh1:
d
UBE2D3_C85A_RR:
UBE2D3_WT_RR:
UBE2D3 sh3:
65
15
e
f
Chromatin
p = 0.0686
p = 0.3811
p = 0.1413
RNF168 level (relative)
0h
3h
6h
24h
Control
Ube2d3 sh1
g
3h 39°C
GFP-RNF168
GFP + dapi
h
Merge
Dapi
γH2AX

Supplementary Figure 5. UBE2D3 counteracts RNF168 accumulation. a, Immunoblots for RNF168 levels upon *UBE2D3* depletion with two independent shRNAs in HeLa and HEK 293T cells. Representative blots from *n=2* independent experiments. **b**, Immunoblots for RNF168 in *Trf2$^{fl/fl}$;Rosa26-CreERT2* MEFs upon 0.6 μM 4-hydroxytamoxifen (4-OHT) treatment and in WT MEFs upon IR. Representative blots from *n=2* independent experiments. **c**, Quantification of exogenously expressed GFP-RNF168 protein levels upon *UBE2D3* depletion in TRF2ts MEFs (representative immunoblot in Fig. 3f). The mean ± SEM from *n=4* independent experiments is shown. Statistical significance was calculated using the two-tailed Student's *t*-test. **d**, Immunoblots for RNF168 in HEK 293T cells with complementation of *UBE2D3* depletion by RNAi-resistant (RR) wild-type UBE2D3 (pCDH_UBE2D3_WT_RR) or UBE2D3 C85A (pCDH_UBE2D3_C85A_RR). Representative blots from *n=4* independent experiments. **e**, Immunoblots for RNF168 levels in TRF2ts MEFs with complementation of *Ube2d3* depletion by RNAi-resistant (RR) wild-type UBE2D3 (LZRS_UBE2D3_WT RR) upon 3 h of telomere uncapping at 39°C. Representative blots from *n=1* experiment. **f**, Quantifications of RNF168 in chromatin extracts in TRF2ts MEFs upon telomere uncapping at 39°C (*n=3* independent experiments; mean ± SEM; two-tailed Student's *t*-test). **g**, Representative images of GFP-RNF168 foci in TRF2ts MEFs used in Fig. 3i, upon telomere uncapping at 39°C for 3 h (*n=2* independent experiments). DNA was stained with DAPI. Original magnifications, X63. Scale bar represents 10 μm. **h**, Representative images of immunofluorescence detection of exogenous GFP-RNF168 and endogenous γH2AX in TRF2ts MEFs transduced as indicated, upon telomere uncapping for at 39°C 3 h. DNA was stained with DAPI. Original magnifications, X63. Scale bar: 10 μm. Images are representative for > 20 cells from *n=1* experiment. Source data are provided as a Source Data file.

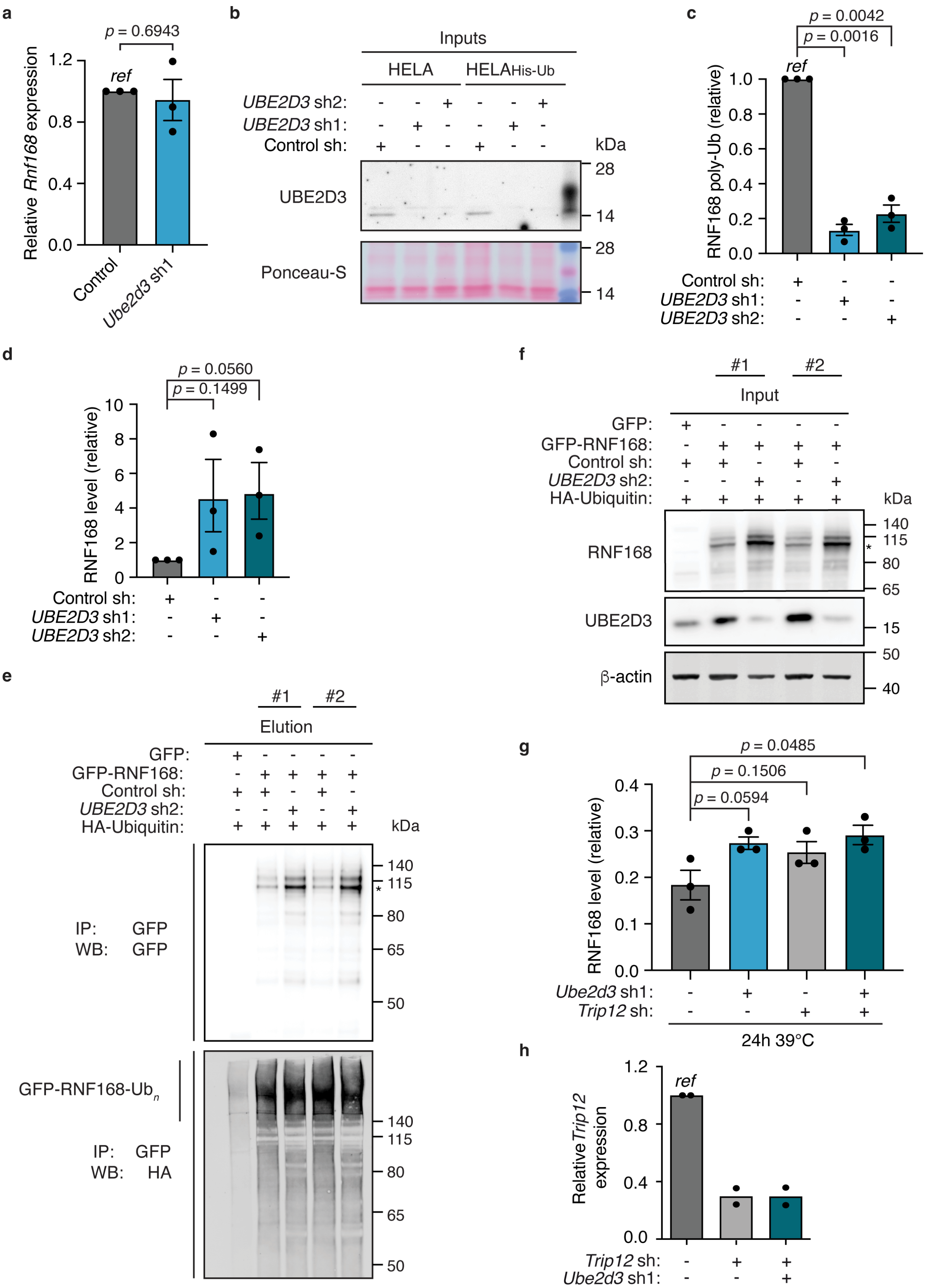
a
p = 0.6943
ref
Relative Rnf168 expression
1.2
0.8
0.4
0.0
Control
Ube2d3 sh1
b
Inputs
HELA
HELAHis-Ub
UBE2D3 sh2:
UBE2D3 sh1:
Control sh:
kDa
UBE2D3
Ponceau-S
28
14
c
p = 0.0042
p = 0.0016
ref
RNF168 poly-Ub (relative)
1.0
0.8
0.6
0.4
0.2
0.0
Control sh:
UBE2D3 sh1:
UBE2D3 sh2:
d
p = 0.0560
p = 0.1499
RNF168 level (relative)
10
8
6
4
2
0
Control sh:
UBE2D3 sh1:
UBE2D3 sh2:
e
#1
#2
Elution
GFP:
GFP-RNF168:
Control sh:
UBE2D3 sh2:
HA-Ubiquitin:
kDa
IP: GFP
WB: GFP
140
115
80
65
50
GFP-RNF168-Ub$_n$
IP: GFP
WB: HA
f
#1
#2
Input
GFP:
GFP-RNF168:
Control sh:
UBE2D3 sh2:
HA-Ubiquitin:
kDa
RNF168
UBE2D3
β-actin
140
115
80
65
15
50
40
g
p = 0.0485
p = 0.1506
p = 0.0594
RNF168 level (relative)
0.4
0.3
0.2
0.1
0.0
Ube2d3 sh1:
Trip12 sh:
24h 39°C
h
ref
Relative Trip12 expression
1.2
0.8
0.4
0.0
Trip12 sh:
Ube2d3 sh1:

Supplementary Figure 6. UBE2D3 restricts RNF168 accumulation and promotes RNF168 ubiquitination without affecting RNF168 mRNA levels. a, q-RT PCR analysis of *Rnf168* mRNA levels in TRF2ts MEFs (*n=3* independent experiments; mean ± SEM; two-tailed Student's *t*-test). **b**, Immunoblots for UBE2D3 to analyse knockdown efficiency in input samples from the IP shown in Fig. 4e. Representative blots from *n=3* independent experiments are shown. **c**,**d**, Quantification of polyubiquitinated RNF168 from the pull down assays in Fig. 4e (**c**) and total RNF168 levels in the input samples in Fig. 4e (**d**). The mean ± SEM from *n=3* independent experiments is shown. Statistical significance was calculated using the two-tailed Student's *t*-test. **e**, Immunoblots for the elution fractions of two independent GFP-IP experiments (#1 and #2) with control sh or *UBE2D3* sh2-transduced 293T cells, transfected with GFP or GFP-RNF168 and HA-ubiquitin. Cells were incubated with 10 μM MG132 for 6 h before harvest. Asterisk indicates GFP-RNF168. **f**, Immunoblots for the input samples of the IPs in **e**, probed with the indicated antibodies. Asterisk indicates GFP-RNF168. **g**, Quantification of RNF168 protein levels upon *Ube2d3* and *Trip12* depletion in TRF2ts MEFs (representative immunoblots in Fig. 4i). The mean ± SEM from *n=3* independent experiments is shown. Statistical significance was calculated using the two-tailed Student's *t*-test. **h**, q-RT PCR analysis of *Trip12* mRNA levels for cells used in Fig. 4i,j (*n=2* independent experiments). *Ref* = reference. Source data are provided as a Source Data file.

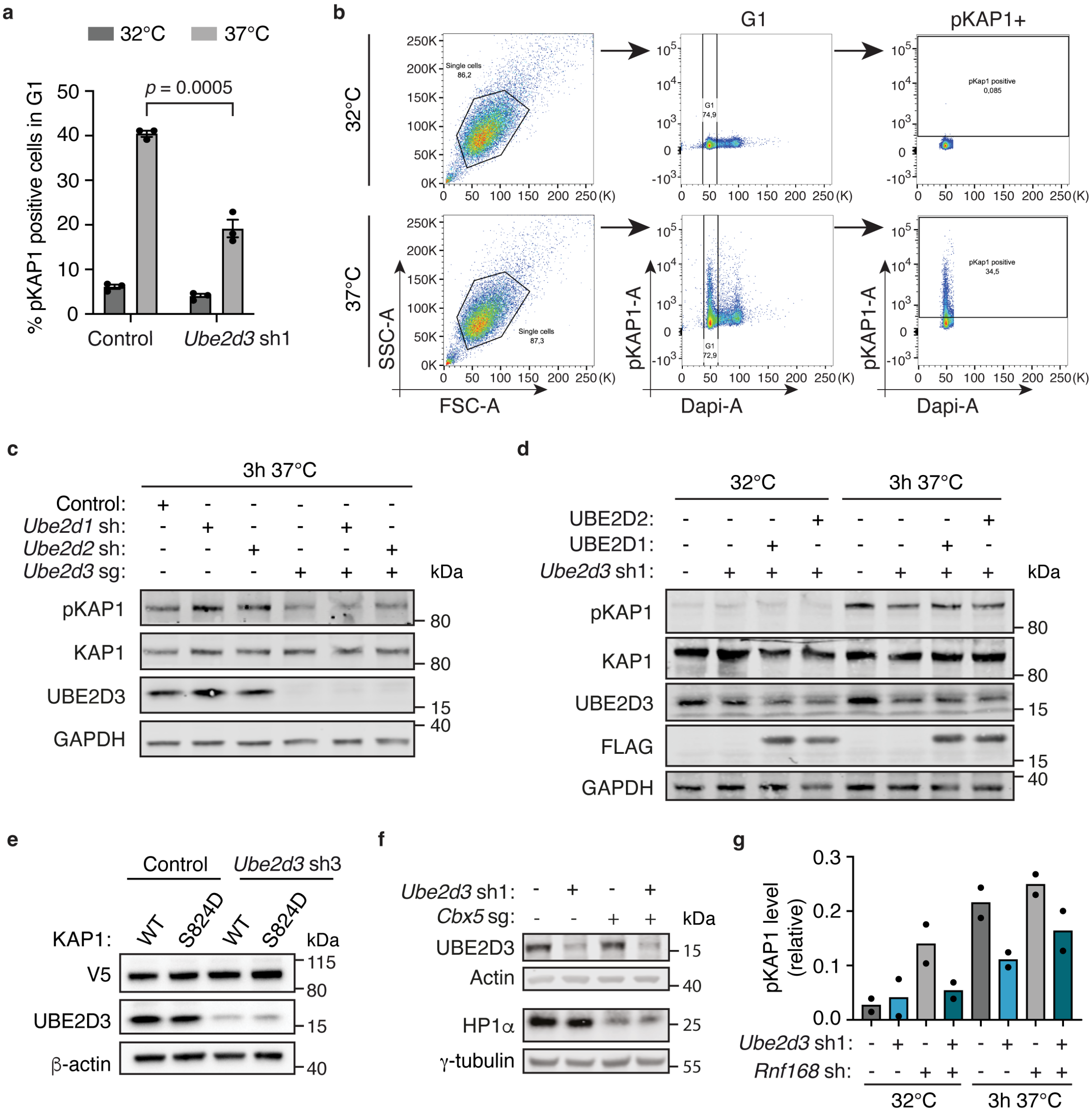
a
32°C
37°C
% pKAP1 positive cells in G1
p = 0.0005
Control
Ube2d3 sh1
b
G1
pKAP1+
32°C
37°C
SSC-A
FSC-A
pKAP1-A
Dapi-A
Single cells
pKap1 positive
c
3h 37°C
Control:
Ube2d1 sh:
Ube2d2 sh:
Ube2d3 sg:
kDa
pKAP1
KAP1
UBE2D3
GAPDH
d
32°C
3h 37°C
UBE2D2:
UBE2D1:
Ube2d3 sh1:
kDa
pKAP1
KAP1
UBE2D3
FLAG
GAPDH
e
Control
Ube2d3 sh3
KAP1:
WT
S824D
kDa
V5
UBE2D3
β-actin
f
Ube2d3 sh1:
Cbx5 sg:
kDa
UBE2D3
Actin
HP1α
γ-tubulin
g
pKAP1 level (relative)
Ube2d3 sh1:
Rnf168 sh:
32°C
3h 37°C

Supplementary Figure 7. UBE2D3 promotes KAP1 phosphorylation non-redundantly with UBE2D1 and UBE2D2 by counteracting RNF168 accumulation and facilitates telomere NHEJ in a pKAP1 and HP1α dependent manner. **a**, Quantification of flow cytometry analysis of the percentage of pKAP1 (S824) positive cells in G1 phase in TRF2ts MEFs transduced with control or *Ube2d3* shRNAs, at 32°C or upon telomere uncapping for 3 h at 37°C. The mean ± SEM from *n=3* independent experiments is shown. Statistical significance was calculated using the two-tailed Student's *t*-test. **b**, Example of gating strategy for pKAP1 positive cells in G1 phase, used in Supplementary Figure 7a. **c**, Immunoblots for pKAP1 levels in TRF2ts MEFs transduced with *Ube2d1* shRNA, *Ube2d2* shRNA and/or *Ube2d3* sgRNA. Representative blots from *n=3* independent experiments. **d**, Immunoblots for pKAP1 levels in TRF2ts MEFs transduced with *Ube2d3* sh1 and complemented with exogenously expressed control, 3xFLAG-UBE2D1 or 3xFLAG-UBE2D2 constructs. Representative blots from *n=3* independent experiments. **e**, Immunoblotting for V5-KAP1 (WT/S824D) and UBE2D3 in TRF2ts MEFs transduced with control or *Ube2d3* shRNA and complemented with exogenously expressed KAP1 WT or S824D mutant, as indicated. Representative blots from *n=3* independent. **f**, Immunoblots for HP1α and UBE2D3 in TRF2ts MEFs from Fig. 5h. Representative blots from *n=3* independent experiments. **g**, Quantification of pKAP1 levels in TRF2ts MEFs transduced with control, *Ube2d3* and/or *Rnf168* shRNAs (*n=2* independent experiments). *Ref* = reference. Source data are provided as a Source Data file.

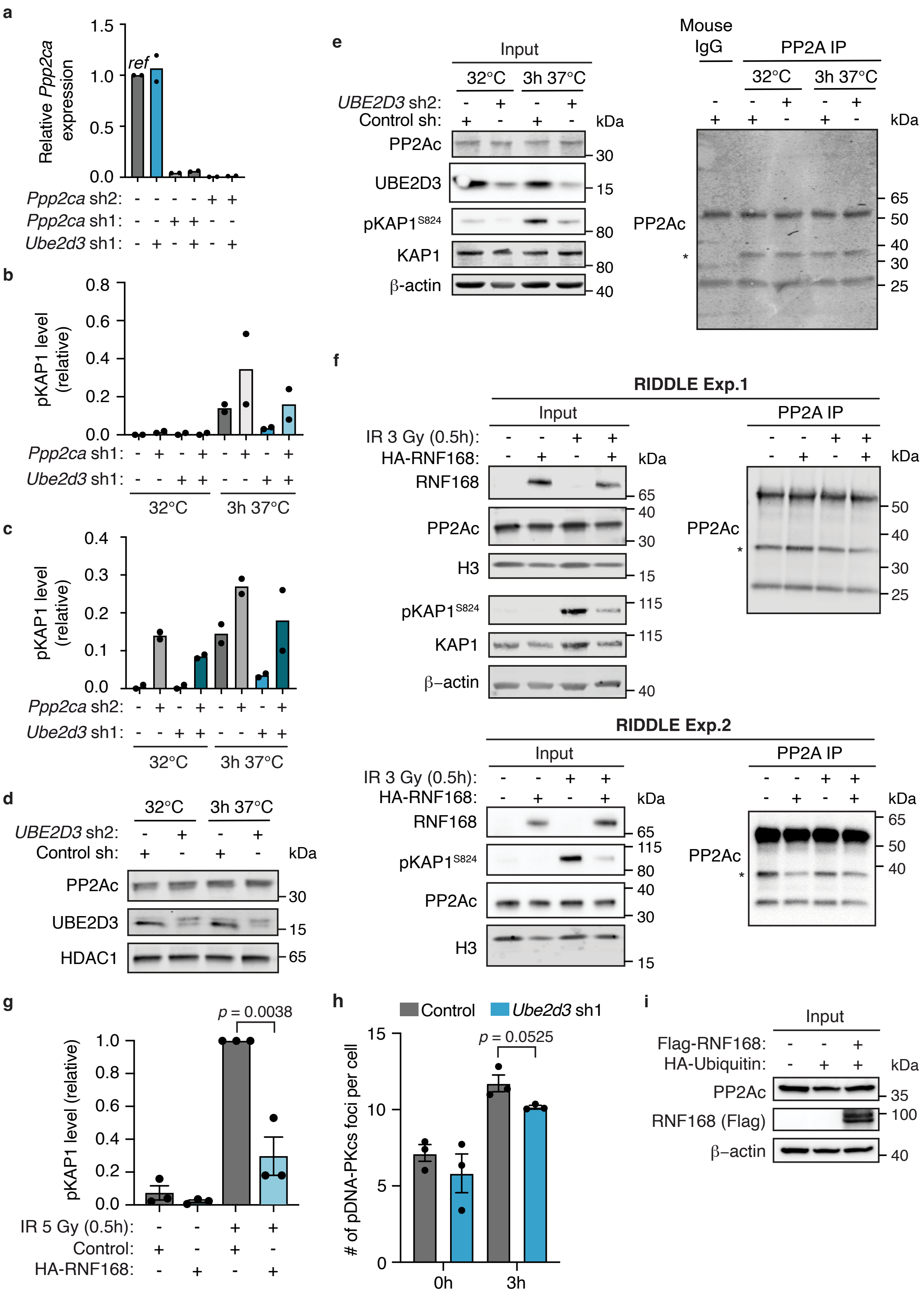

a
Relative Ppp2ca expression
1.5
1.0
0.5
0.0
ref
Ppp2ca sh2: - - - - + +
Ppp2ca sh1: - - + + - -
Ube2d3 sh1: - + - + - +
b
pKAP1 level (relative)
0.8
0.6
0.4
0.2
0.0
Ppp2ca sh1: - + - + - + - +
Ube2d3 sh1: - - + + - - + +
32°C
3h 37°C
c
pKAP1 level (relative)
0.4
0.3
0.2
0.1
0.0
Ppp2ca sh2: - + - + - + - +
Ube2d3 sh1: - - + + - - + +
32°C
3h 37°C
d
32°C
3h 37°C
UBE2D3 sh2: - + - +
Control sh: + - + -
kDa
PP2Ac
UBE2D3
HDAC1
30
15
65
e
Input
32°C
3h 37°C
UBE2D3 sh2: - + - +
Control sh: + - + -
kDa
PP2Ac
UBE2D3
pKAP1S824
KAP1
β-actin
30
15
80
80
40
Mouse IgG
PP2A IP
32°C
3h 37°C
- - + - +
+ + - + -
kDa
PP2Ac
*
65
50
40
30
25
f
RIDDLE Exp.1
Input
PP2A IP
IR 3 Gy (0.5h): - - + +
HA-RNF168: - + - +
kDa
RNF168
PP2Ac
H3
pKAP1S824
KAP1
β-actin
65
40
30
15
115
115
40
PP2Ac
*
50
40
30
25
RIDDLE Exp.2
Input
PP2A IP
IR 3 Gy (0.5h): - - + +
HA-RNF168: - + - +
kDa
RNF168
pKAP1S824
PP2Ac
H3
65
115
80
40
30
15
PP2Ac
*
65
50
40
g
pKAP1 level (relative)
p = 0.0038
1.0
0.8
0.6
0.4
0.2
0.0
IR 5 Gy (0.5h): - - + +
Control: + - + -
HA-RNF168: - + - +
h
Control
Ube2d3 sh1
# of pDNA-PKcs foci per cell
p = 0.0525
15
10
5
0
0h
3h
i
Input
Flag-RNF168: - - +
HA-Ubiquitin: - + +
kDa
PP2Ac
RNF168 (Flag)
β-actin
35
100
40

Supplementary Figure 8. UBE2D3 depletion or ectopic RNF168 expression enhance PP2A activity and suppress KAP1-S824 phosphorylation, and UBE2D3 promotes DNA-PKcs phosphorylation. **a**, q-RT PCR analysis of *Ppp2ca* mRNA levels in TRF2ts MEFs used in Fig. 6a,b (*n=2* independent experiments). **b**,**c**, Quantification of pKAP1 levels in TRF2ts MEFs upon *Ube2d3* and/or *Ppp2ca* depletion and telomere uncapping at 37°C (**b**: *Ppp2ca* sh1, **c**: *Ppp2ca* sh2; *n=2* independent experiments). **d**, Immunoblotting for PP2A catalytic subunit PP2A-alpha (PP2Ac) in TRF2ts MEF transduced as indicated upon telomere uncapping for 3 h at 37°C. Representative blots from *n=3* independent experiments. **e**, Left: Immunoblotting for PP2A-alpha (PP2Ac), UBE2D3, pKAP1-S824 and total KAP1 in the input samples used for PP2A phosphatase activity assays (Fig. 6c). Right: Immunoblotting for PP2A-alpha (PP2Ac) in the PP2A IP fraction samples of the PP2A activity assays in Fig. 6c. **f**, Left: Immunoblotting for RNF168, PP2A-alpha (PP2Ac) and pKAP1-S824 in the input samples used for PP2A phosphatase activity assays in RIDDLE cells (Fig. 6d). Right: Immunoblotting for PP2A-alpha (PP2Ac) in the PP2A IP fraction samples of the PP2A activity assays in Fig. 6d. **g**, Quantification of pKAP1 levels in RIDDLE cells +/- HA-RNF168 (representative immunoblots in Supplementary Figure 8f). *N=3* independent experiments are shown. Statistical significance was calculated using the two-tailed Student's *t*-test. **h**, Quantification of average number of pDNA-PKcs foci in TRF2ts MEFs, transduced as indicated, at 32°C (0 h) or upon telomere uncapping for 3 h at 39°C (*n=3* independent experiments; mean ± SEM; two-tailed Student's *t*-test). **i**, Immunoblots for PP2Ac and Flag-RNF168 in the input samples used for *in vivo* ubiquitination of endogenous PP2A catalytic subunit C by RNF168 in Fig. 6e. Representative blots from *n=3* independent experiments. *Ref* = reference. Source data are provided as a Source Data file.

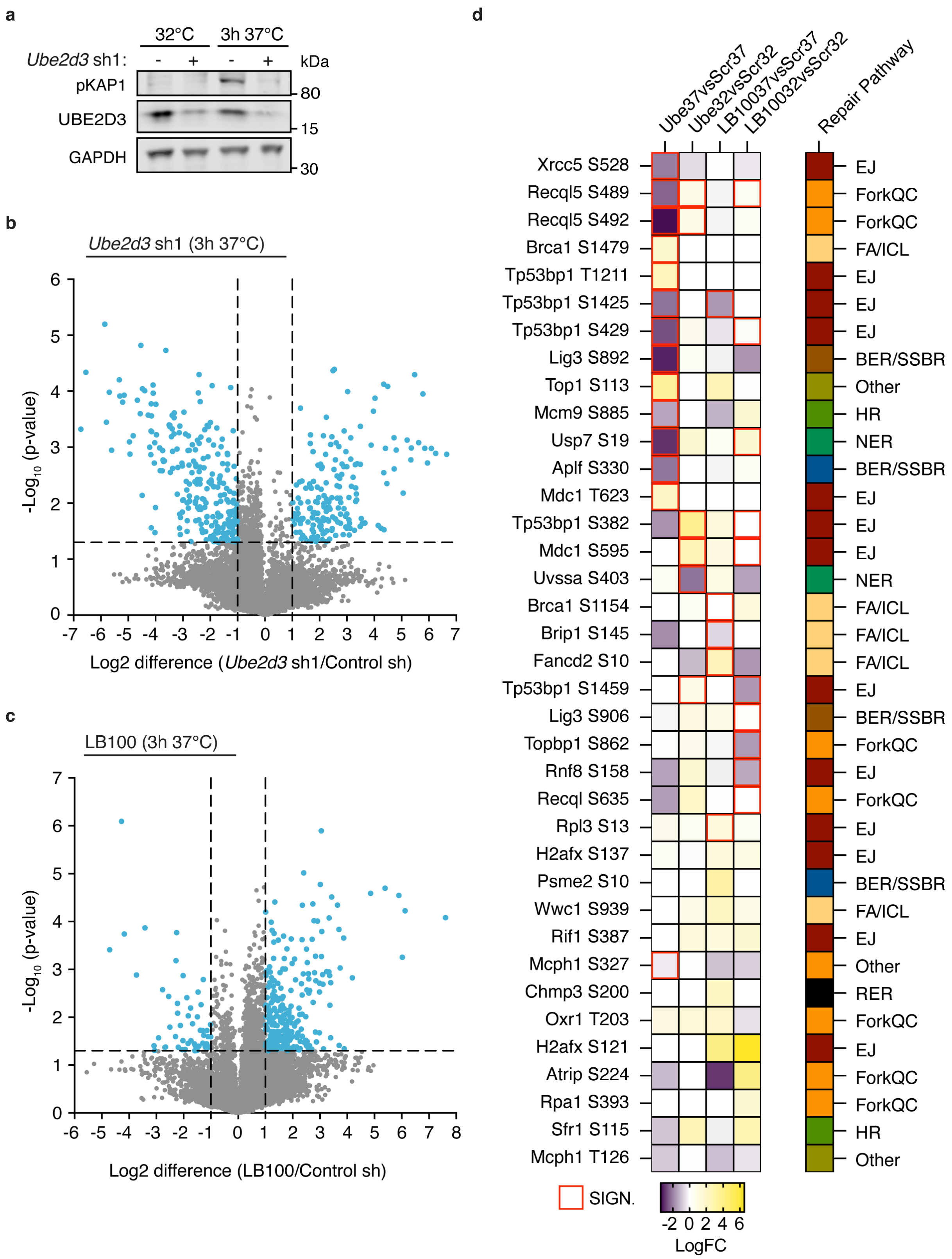
a
32°C
3h 37°C
Ube2d3 sh1:
kDa
pKAP1
UBE2D3
GAPDH
80
15
30
b
Ube2d3 sh1 (3h 37°C)
-Log10 (p-value)
Log2 difference (Ube2d3 sh1/Control sh)
c
LB100 (3h 37°C)
-Log10 (p-value)
Log2 difference (LB100/Control sh)
d
Ube37vsScr37
Ube32vsScr32
LB10037vsScr37
LB10032vsScr32
Repair Pathway
Xrcc5 S528
Recql5 S489
Recql5 S492
Brca1 S1479
Tp53bp1 T1211
Tp53bp1 S1425
Tp53bp1 S429
Lig3 S892
Top1 S113
Mcm9 S885
Usp7 S19
Aplf S330
Mdc1 T623
Tp53bp1 S382
Mdc1 S595
Uvssa S403
Brca1 S1154
Brip1 S145
Fancd2 S10
Tp53bp1 S1459
Lig3 S906
Topbp1 S862
Rnf8 S158
Recql S635
Rpl3 S13
H2afx S137
Psme2 S10
Wwc1 S939
Rif1 S387
Mcph1 S327
Chmp3 S200
Oxr1 T203
H2afx S121
Atrip S224
Rpa1 S393
Sfr1 S115
Mcph1 T126
EJ
ForkQC
ForkQC
FA/ICL
EJ
EJ
EJ
BER/SSBR
Other
HR
NER
BER/SSBR
EJ
EJ
EJ
NER
FA/ICL
FA/ICL
FA/ICL
EJ
BER/SSBR
ForkQC
EJ
ForkQC
EJ
EJ
BER/SSBR
FA/ICL
EJ
Other
RER
ForkQC
EJ
ForkQC
ForkQC
HR
Other
SIGN.
LogFC

Supplementary Figure 9. Phosphoproteome analysis of UBE2D3-depleted or PP2A inhibitor-treated cells in the presence or absence of telomere uncapping. a, Immunoblotting for UBE2D3 and pKAP1 in TRF2ts MEFs used for phosphoproteomics experiments. Representative blots from *n=3* independent experiments. **b**, Volcano plot showing the log2 fold changes of phosphorylated peptides in phosphoproteome analysis upon UBE2D3 depletion with shRNA1 in TRF2ts MEFs upon 3 h of telomere uncapping at 37°C (*n=3* independent experiments). Blue dots represent peptides that are significantly enriched (at least 2x upregulated or downregulated in their phosphorylation (log2 ≥ 1.0 or log2 ≤ –1.0) and have a *p* value of ≤ 0.05. **c**, Volcano plot showing the log2 fold changes of phosphorylated peptides in phosphoproteome analysis upon treatment with LB100 (PP2A-inhibitor; 5 μM for 15 h) of TRF2ts MEFs upon 3 h of telomere uncapping at 37°C (*n=3* independent experiments). Blue dots represent peptides that are significantly enriched (at least 2x upregulated or downregulated in their phosphorylation (log2 ≥ 1.0 or log2 ≤ –1.0) and have a *p* value of ≤ 0.05. **d**, Left: Heatmap of phosphorylation levels of indicated phosphosites of DNA repair factors in respective pairwise comparisons. Red squares indicate significance (SIGN; *p* ≤ 0.05) Right: DNA repair factors were mapped to respective repair pathways according to classification used before[1]**.** Colors represent the different repair pathways. LogFC = Log fold change. Source data are provided as a Source Data file.

Supplementary References

1. Olivieri, M. et al. A Genetic Map of the Response to DNA Damage in Human Cells. Cell 182, 481-496 e421 (2020). https://doi.org:10.1016/j.cell.2020.05.040
