# Supplementary material for: UBE2D3 facilitates NHEJ by orchestrating ATM signalling through multi-level control of RNF168
Source: Nat Commun. 2024 Jun 12;15:5032. doi: 10.1038/s41467-024-49431-6 (PMC11169547; doi:10.1038/s41467-024-49431-6)
Supplement: Supplementary file 3 — Reporting Summary [file 41467_2024_49431_MOESM3_ESM.pdf]

nature portfolio

Corresponding author(s): JJL Jacobs

Last updated by author(s): May 7, 2024

Reporting Summary

Nature Portfolio wishes to improve the reproducibility of the work that we publish. This form provides structure for consistency and transparency in reporting. For further information on Nature Portfolio policies, see our Editorial Policies and the Editorial Policy Checklist.

Statistics

For all statistical analyses, confirm that the following items are present in the figure legend, table legend, main text, or Methods section.

n/a	Confirmed	
☐	☒	The exact sample size (*n*) for each experimental group/condition, given as a discrete number and unit of measurement
☐	☒	A statement on whether measurements were taken from distinct samples or whether the same sample was measured repeatedly
☐	☒	The statistical test(s) used AND whether they are one- or two-sided<br>*Only common tests should be described solely by name; describe more complex techniques in the Methods section.*
☒	☐	A description of all covariates tested
☐	☒	A description of any assumptions or corrections, such as tests of normality and adjustment for multiple comparisons
☐	☒	A full description of the statistical parameters including central tendency (e.g. means) or other basic estimates (e.g. regression coefficient) AND variation (e.g. standard deviation) or associated estimates of uncertainty (e.g. confidence intervals)
☐	☒	For null hypothesis testing, the test statistic (e.g. *F*, *t*, *r*) with confidence intervals, effect sizes, degrees of freedom and *P* value noted<br>*Give P values as exact values whenever suitable.*
☒	☐	For Bayesian analysis, information on the choice of priors and Markov chain Monte Carlo settings
☒	☐	For hierarchical and complex designs, identification of the appropriate level for tests and full reporting of outcomes
☒	☐	Estimates of effect sizes (e.g. Cohen's *d*, Pearson's *r*), indicating how they were calculated

Our web collection on statistics for biologists contains articles on many of the points above.

Software and code

Policy information about availability of computer code

Data collection	1. Syngene G:BOX (Syngene)<br>2. Odyssey Infrared imager (LI-COR)<br>3. Chemidoc XRS+ 4.0.1 (Bio-Rad)<br>4. Metafer4/MSearch (Metasystems) on AxioImagerZ2 microscope (Carl Zeiss)<br>5. Leica SP5 (Leica Microsystems)<br>6. FACS Calibur (BD Biosciences)<br>7. Fortessa analyser (BD Biosciences)<br>8. Infinite M200pro (TECAN)<br>9. StepOnePlus real-time PCR system (Applied Biosystems)<br>10. LightCycler 480 II (Roche)<br>11. Bioruptor (Diagenode)<br>12. CHEF-DR III pulsed-field gel electrophoresis system (Bio-Rad)<br>13. Col-Count (Oxford Optronix)<br>14. Fujifilm FLA-3000 R laser imaging scanner<br>15. Orbitrap Exploris 480 Mass Spectrometer (Thermo)
Data analysis	1. Syngene G:BOX (Genesys software version 1.6.9.0)<br>2. Fiji/ImageJ (version 1.52p)<br>3. LAS-AF (version 2.7.4)<br>4. FlowJo (version 10)<br>5. GraphPad Prism (version 9.0.0)<br>6. StepOnePlus real-time PCR system (software version 2.2.2.)

7. LightCycler 480 II (software version 1.5.0 sp3)
8. ImageStudio (version 5.2.5)
9. Image Lab (version 6.0.1)
10. AIDA Image Analyzer software (version 3.40)
11. Col-Count (version 3.3)
12. Perseus (version 2.0.10.0)
13. MaxQuant (version 2.4.2.0)
14. DIA-NN (version 1.8)

For manuscripts utilizing custom algorithms or software that are central to the research but not yet described in published literature, software must be made available to editors and reviewers. We strongly encourage code deposition in a community repository (e.g. GitHub). See the Nature Portfolio guidelines for submitting code & software for further information.

Data

Policy information about availability of data

All manuscripts must include a data availability statement. This statement should provide the following information, where applicable:

- Accession codes, unique identifiers, or web links for publicly available datasets
- A description of any restrictions on data availability
- For clinical datasets or third party data, please ensure that the statement adheres to our policy

Source data are provided with this paper. The mass spectrometry proteomics data generated in this study have been deposited in the ProteomeXchange Consortium via the PRIDE62 partner repository under accession code PXD044410 [https://proteomecentral.proteomexchange.org/cgi/GetDataset?ID=PXD044410]. All other relevant data are available from the corresponding author upon request. All data generated this study are included in this published article and its supplementary information files, and are available from the corresponding author upon request.

Field-specific reporting

Please select the one below that is the best fit for your research. If you are not sure, read the appropriate sections before making your selection.

☒ Life sciences ☐ Behavioural & social sciences ☐ Ecological, evolutionary & environmental sciences

For a reference copy of the document with all sections, see nature.com/documents/nr-reporting-summary-flat.pdf

Life sciences study design

All studies must disclose on these points even when the disclosure is negative.

Sample size	No sample size calculations were done. The number of replicates is mentioned in the figure legends and the corresponding data point for each individual biological replicate is shown in the graphs.<br>1. For metaphase chromosome fusion analysis, we analyzed >30 metaphase spreads per experiment to get to a total of at least 1500 chromosomes per condition within each biological replicate. Analysis of >1000 chromosomes per condition and replicate is sufficient to obtain a reliable mean.<br>2. For immunofluorescence experiments we acquired a minimum of 100 cells per condition per experiment. Analysis of >100 cells was enough to obtain a representative mean of the number of foci of an experimental condition.
Data exclusions	No samples or data points were excluded from individual experiments. In the rare event that internal negative or positive controls within an individual experiment did not perform as expected, this entire individual experiment (with all samples) was discarded.
Replication	Most of the experiments contain internal controls that have been validated before. Experiments were performed at least in duplicate, but mostly in triplicate or more to assess the reproducibility. Graphs are represented as dot plots showing the spread between the replicates of the individual experiments. Standard errors are included in the graphs. The number of replicates for each experiment is mentioned in the figure legends. For the experiment shown in Supplementary Fig. 2c "BJ", the dots represent individual metaphase spreads within the conditions.
Randomization	No randomization was used. Experiments were performed with cell lines that were seeded and analyzed randomly for different treatments.
Blinding	For metaphase spread analysis, generally genotypes were blinded before scoring in 1-2 out of the total number of replicates (Fig. 2a-c, Fig. 4g, Fig. 5g,h, Fig. 6b). For immunofluorescence assays, all foci were counted and analyzed either by Metafer software when images were captured using the Metafer platform, or by an ImageJ macro developed in house when images were acquired with a confocal microscope. Therefore, no manual counting was done for foci analysis, except for the 53BP1 foci upon 3h of telomere uncapping in Fig. 3b. For many other approaches used in the manuscript, including western blots and IPs, blinding was not feasible.

Reporting for specific materials, systems and methods

We require information from authors about some types of materials, experimental systems and methods used in many studies. Here, indicate whether each material, system or method listed is relevant to your study. If you are not sure if a list item applies to your research, read the appropriate section before selecting a response.

Materials & experimental systems

n/a	Involved in the study
	☒ Antibodies
	☒ Eukaryotic cell lines
☒	Palaeontology and archaeology
☒	Animals and other organisms
☒	Human research participants
☒	Clinical data
☒	Dual use research of concern

Methods

n/a	Involved in the study
☒	ChIP-seq
	☒ Flow cytometry
☒	MRI-based neuroimaging

Antibodies

Antibodies used

For an overview of antibodies used, see Methods section.
Primary antibodies:
1. UBE2D3 Y-25, sc-100618, Santa-Cruz, 1:500 (Figure 1b)
2. UBE2D3, 11677-1-AP, Proteintech, 1:500 (Fig. 2h and Supplementary Fig. 1f)
3. UBE2D3 4330S, CST, 1:500 (Fig. 1c, 2c, 4i, 5e and Supplementary Fig. 1a, 5d, 6b, 7c, 7d, 7e,7f, 8d, 8e, 9a)
4. UBCH5, 615, Boston Biochem, 1:2,000 (Fig. 2d, 3a, 3b, 3c, 3d, 3f,3h, 4a, 4c, 4d, 4h, 5a, 5i, 6a and Supplementary Fig. 2b, 2e, 5a, 5b, 5e, 6f)
5. KAP1, 22553, Abcam, 1:1,000
6. phospho-Kap1 S824, A300 767A, Bethyl, 1:1,000
7. 53BP1, NB100-305, Novus, 1:500
8. 53BP1, A300-272A, Bethyl, 1:2,000
9. phospho-ATM S1981, 4526, CST, 1:1,000
10. phospho-H2AX S139, 5636, Millipore, 1:1,000
11. CHK2, 611570, BD, 1:500
12. c-myc 9E10, sc-40, Santa-Cruz, 1:250
13. HA, MMS-101R, Covance, 1:1,000
14. TRF2, NB110-57130, Novus, 1:500
15. RNF8, sc-133971, Santa-Cruz, 1:250
16. MAD2L2, sc-135977, Santa-Cruz, 1:500
17. GFP IgG fraction, A11122, ThermoFisher Scientific, 1:1,000
18. Histone H3, ab1791, Abcam, 1:10,000
19. hRNF168, ABE367, Millipore, 1:500
20. hRNF168, ABE467, Merck-Millipore, 1:1,000
21. mRnf168, gift from D. Durocher, 1:1,000
22. hRIF1, A300-569A, Bethyl, 1:1,000
23. mRIF1, gift from S. Boulton and R. Chapman, 1:1,000
24. Ligase 4 H-300, sc-28232, Santa-Cruz, 1:300
25. FK2, 04-263, Millipore, 1:2,000
26. Ubiquitin P4D1, sc-8017, Santa-Cruz, 1:1,000
27. HDAC1, PA1-860, ThermoFisher Scientific, 1:1,000
28. PP2A C subunit, clone 1D6 antibody, 05-421, Sigma Aldrich/Millipore, 1:500
29. HSP90 H-114, sc-7947, Santa-Cruz, 1:1,000
30. gamma-tubulin, T6557, Sigma, 1:10,000
31. beta-actin, A5316, Sigma, 1:10,000
32. beta-catenin, 610154, BD, 1:10,000
33. phospho-DNA PKcs S2056, ab18192, Abcam, 1:500
34. HP1 alpha, 2616S, CST, 1:1000
35. Ligase IV, NB110-57379, Novus, 1:500
36. GAPDH, PA1-987, ThermoFisher Scientific, 1:1000
37. Flag M2, F1804, Sigma, 1:1000

Secondary antibodies:
1. Alexa Fluor 488 goat anti-mouse IgG A11029, Invitrogen, 1:500
2. Alexa Fluor 488 goat anti-rabbit IgG A11008, Invitrogen, 1:500
3. Alexa Fluor 568 goat anti-mouse IgG A11031, Invitrogen, 1:500
4. Alexa Fluor 568 goat anti-rabbit IgG A211011, Invitrogen, 1:500
5. Goat anti-rabbit IgG HRP, G21234, Invitrogen, 1:7,500
6. Goat anti-mouse IgG HRP, G21040, Invitrogen, 1:7,500
7. IRDye800CW Goat anti-mouse IgG, 926-32210, LI-COR, 1:10,000
8. IRDye800CW Goat anti-rabbit IgG, 926-32211, LI-COR, 1:10,000

9. IRDye680 Goat anti-mouse IgG, 926-32220, LI-COR, 1:10,000
10. IRDye680 Goat anti-rabbit IgG, 926-32221, LI-COR, 1:10,000

Validation

Commercially available antibodies were validated by the manufacturer/supplier.

The following antibodies were additionally validated by us:
-In knockdown cells: UBE2D3 Y-25 (sc-100618, Santa-Cruz), UBE2D3 (11677-1-AP, Proteintech), UBE2D3 (4330S, CST), UBCH5 (615, Boston Biochem), hRIF1 (A300-569A, Bethyl), 53BP1 (A300-272A, Bethyl) and mRIF1 (gift from S. Boulton and R. Chapman).
-In Western blot analysis to detect DNA damage-induced phosphorylation: phospho-Kap1 S824 (A300-767A, Bethyl)
-IP experiments: GFP (A11122, Life Technologies) and PP2A C subunit, clone 1D6 (05-421, Sigma Aldrich/Millipore)
-DNA damaged-induced foci: GFP (A11122, Life Technologies), phospho-H2AX S139 (5636, Millipore) and phospho-ATM S1981 (4526, CST).

The following antibodies are commonly used loading controls: HSP90 (sc7947, Santa Cruz), gamma-tubulin (T6557, Sigma), H3 (ab1791, Abcam), beta-actin (A5316, Sigma) and beta-catenin (610154, BD)

Eukaryotic cell lines

Policy information about cell lines

Cell line source(s)

1. HeLa, U2OS, BJ, HEK-293T and Phoenix originate from ATCC.
2. TRF2ts MEFs are described before (Konishi et al, Genes & Development, 2008, Peuscher et al, Nature cell biology, 2011).
3. Dox-inducible shTRF2 HeLa cells are gifted from Joachim Lingner (EPFL). Cell line was described before (Grolimund et al, Nature Communications, 2013).

Authentication

None of the cell lines used were authenticated by us.

Mycoplasma contamination

All cell lines are routinely tested negative for mycoplasma contamination.

Commonly misidentified lines
(See ICLAC register)

No commonly misidentified cell lines were used in this study.

Flow Cytometry

Plots

Confirm that:

- [x] The axis labels state the marker and fluorochrome used (e.g. CD4-FITC).
- [x] The axis scales are clearly visible. Include numbers along axes only for bottom left plot of group (a 'group' is an analysis of identical markers).
- [x] All plots are contour plots with outliers or pseudocolor plots.
- [x] A numerical value for number of cells or percentage (with statistics) is provided.

Methodology

Sample preparation

Sample preparation is specified in the Methods section.
1. For aneuploidy (4N) analysis, cells were collected by trypsinization, fixed and stained with propidium iodide (PI), followed by flow cytometry analysis.
2. For cell cycle distribution analysis, cells were treated with BrdU, trypsinized, fixed and stained with anti-BrdU antibody prior to staining with FITC-conjugated goat anti-mouse secondary antibody. Subsequently, cells were stained with propidium iodide (PI) and analyzed by flow cytometry analysis.
3. For pKAP1, cells were collected by trypsinization, fixed and stained with phospho-Kap1 S824 antibody prior to staining with Alexa Fluor 647 goat anti-rabbit secondary antibody and subsequently stained with dapi, followed by flow cytometry analysis.

Instrument

1. Fortessa analyser (BD Biosciences)
2. FACSCalibur (BD Biosciences)

Software

FlowJo (version 10)

Cell population abundance

No cell sorting was used.

Gating strategy

1. For aneuploidy analysis, cells were gated for live cells (FSC-H/SSC-H) and analyzed for PI to address DNA-content.
2. For cell cycle distribution analysis, cells were first gated for live cells (FSC-A/SSC-A). Next, cells were gated for single cells (610_20-W/610_20-A) and analyzed for PI and FITC (BrdU) fluorescence to identify the proportion of cells in each cell cycle phase.
3. For pKAP1 analysis, cells were initially gated for single cells (FSC-A/SSC-A). Then, dapi staining was used to gate for cells in G1 and pKAP1-positive cells were quantified.

- [x] Tick this box to confirm that a figure exemplifying the gating strategy is provided in the Supplementary Information.
